# Base Pairing Interaction between 5′- and 3′-UTRs Controls *icaR* mRNA Translation in *Staphylococcus aureus*

**DOI:** 10.1371/journal.pgen.1004001

**Published:** 2013-12-19

**Authors:** Igor Ruiz de los Mozos, Marta Vergara-Irigaray, Victor Segura, Maite Villanueva, Nerea Bitarte, Margarida Saramago, Susana Domingues, Cecilia M. Arraiano, Pierre Fechter, Pascale Romby, Jaione Valle, Cristina Solano, Iñigo Lasa, Alejandro Toledo-Arana

**Affiliations:** 1 Laboratory of Microbial Biofilms. Instituto de Agrobiotecnología (IDAB). Universidad Pública de Navarra-CSIC-Gobierno de Navarra. Campus de Arrosadía. Pamplona, Spain; 2 Genomics, Proteomics and Bioinformatics Unit. Center for Applied Medical Research. University of Navarra. Pamplona, Spain; 3 Instituto de Tecnologia Química e Biológica, Universidade Nova de Lisboa. Oeiras, Portugal; 4 Architecture et Réactivité de l'ARN, Université de Strasbourg, CNRS, IBMC. Strasbourg, France; Universidad de Sevilla, Spain

## Abstract

The presence of regulatory sequences in the 3′ untranslated region (3′-UTR) of eukaryotic mRNAs controlling RNA stability and translation efficiency is widely recognized. In contrast, the relevance of 3′-UTRs in bacterial mRNA functionality has been disregarded. Here, we report evidences showing that around one-third of the mapped mRNAs of the major human pathogen *Staphylococcus aureus* carry 3′-UTRs longer than 100-nt and thus, potential regulatory functions. We selected the long 3′-UTR of *icaR*, which codes for the repressor of the main exopolysaccharidic compound of the *S. aureus* biofilm matrix, to evaluate the role that 3′-UTRs may play in controlling mRNA expression. We showed that base pairing between the 3′-UTR and the Shine-Dalgarno (SD) region of *icaR* mRNA interferes with the translation initiation complex and generates a double-stranded substrate for RNase III. Deletion or substitution of the motif (UCCCCUG) within *icaR* 3′-UTR was sufficient to abolish this interaction and resulted in the accumulation of IcaR repressor and inhibition of biofilm development. Our findings provide a singular example of a new potential post-transcriptional regulatory mechanism to modulate bacterial gene expression through the interaction of a 3′-UTR with the 5′-UTR of the same mRNA.

## Author Summary

At both sides of the protein-coding region, the mRNA molecule contains sequences that are not translated to protein. In eukaryotes, the untranslated 3′ region (3′-UTR), which comprises from the last codon used in translation to the 3′ end of the mRNA, controls mRNA stability, location and translation efficiency. In contrast, knowledge about the functions of 3′-UTRs in bacterial physiology is scarce. Here, we demonstrate that bacterial 3′-UTRs might play regulatory functions that might resemble those already described in eukaryotes. Transcriptome analysis of the human pathogen *Staphylococcus aureus* revealed that at least 30% of mRNAs contain long 3′-UTRs. Using the 3′-UTR of the mRNA encoding the main biofilm repressor IcaR as a model, we show that the 3′-UTR interferes with the translation initiation complex and promotes mRNA decay through base pairing with the ribosome binding site. This event contributes to adjusting IcaR level and modulating exopolysaccharide production and biofilm development in *S. aureus*. Our data illustrate that bacterial 3′-UTRs can provide strategies for fine-tuning control of gene expression.

## Introduction

Regulation of translation is used to modulate gene expression in a wide range of biological situations in all living organisms. Compared to transcriptional regulation, mRNA translational control provides several advantages such as a more rapid response, reversibility, fine-tuning of protein amount, coordinated regulation of protein families, potential for spatial control and efficacy in systems lacking transcriptional control mechanisms [1]. In eukaryotes, translational control is largely conferred through specific *cis*-acting sequences located in mRNA 3′ untranslated regions (3′-UTR) that serve as binding sites for associated *trans*-acting factors. These localized 3′-UTR *cis*-acting sequences include microRNAs (miRNAs) specific binding sites, denoted as “seed sequences” that can cause gene silencing by destabilization of target RNAs. miRNAs can also affect the translation process [2]. In addition, the poly(A) tail acts as a binding site for a class of regulatory factors required for some mRNAs to be exported from the nucleus, promotes translation initiation and termination and recycling of ribosomes and enhances stability of mRNA [3], [4]. Furthermore, specific sequence or structure elements are also recognized by RNA-binding proteins or non-coding RNAs, that can either upregulate or downregulate gene expression (for review see [5]). Remarkably, several of these *cis*-acting sequences and *trans*-acting factors have been involved in end-to-end interactions of mRNA (“closed-loop” or “circular” mRNA structure) by which translation can be controlled [1].

In contrast, as regards bacterial translational control, it is mainly modulated through the mRNA 5′-UTR which includes the Shine and Dalgarno (SD) sequence [6]. Bacterial 5′-UTRs also carry secondary structures, binding sites for small regulatory RNAs (sRNAs) or RNA binding proteins that modify mRNA stability or protein translation [7]–[12]. In addition, thermosensors and riboswitches control protein expression through conformational changes upon a temperature shift or ligand binding, respectively [13]–[16]. With respect to the bacterial 3′-UTR, the intrinsic transcriptional terminator sequence which folds in a stem loop secondary structure prevents exonucleases access to the 3′-end of the transcript [17], [18]. Remarkably, recent studies suggest that bacterial 3′-UTRs might have further functions [19]–[23]. For example, the existence of a conserved 3′-UTR in nine membrane-associated genes with conserved long 3′-UTRs in *Bacillus subtilis* suggested a functional role for these 3′-UTRs [19]. In *Listeria monocytogenes* and *Staphylococcus aureus*, long 3′-UTRs that overlap adjacent convergent transcripts encoded at the opposite DNA strand have been described [20], [22]. These overlapping 3′-UTRs may modulate the expression of neighbouring genes by a *cis*-acting antisense RNA mechanism [22], [23]. In addition, 3′-UTRs could act as reservoirs of small regulatory RNAs either by processing the long 3′-UTR or by *de novo* transcription from an internal promoter [24], [25]. Finally, co-immunoprecipitation experiments have shown a high affinity of *Salmonella typhimurium* Hfq and *S. aureus* RNase III proteins for mRNA 3′-UTRs suggesting that this region may provide a regulatory function [26], [27].

In this study, we mapped the 3′ boundaries of the *S. aureus* transcriptome combining custom-tiling microarrays and directional RNA-deep sequencing data. Results uncovered that at least one third of the *S. aureus* transcripts carry 3′-UTRs longer than 100 nucleotides (nt). Since this 3′-UTR length provides significant potential for transcript-specific regulation, we examined the putative role of bacterial 3′-UTRs in gene regulation using the long 3′-UTR of *icaR* transcript as a model. We chose *icaR* because of its involvement in the regulation of biofilm formation. This process is the main cause of nosocomial infections in patients with indwelling medical devices [28]. Bacteria in a biofilm are surrounded by a self-produced extracellular matrix that contains exopolysaccharides, proteins and sometimes DNA. In the case of *S. aureus*, the main biofilm exopolysaccharide is a poly-β-1,6-*N*-acetylglucosamine polymer (PIA-PNAG), whose synthesis depends on the enzymes encoded by the *icaADBC* operon [29], [30]. One of the regulators that controls *icaADBC* expression is IcaR, a member of the TetR family of transcriptional regulatory proteins. IcaR is encoded at the *ica* locus but is divergently transcribed from the *icaADBC* operon. Binding of IcaR to the *icaADBC* promoter inhibits *icaADBC* expression [31]. As regards the putative regulatory elements modulating IcaR levels, they remain poorly understood.

Here, we first showed that deletion of the 3′-UTR of *icaR* caused a stabilization of *icaR* mRNA and consequently an increase in IcaR protein levels, indicating that *icaR* 3′-UTR affects mRNA half-life. Then, *icaR* mRNA secondary structure prediction showed that a UCCCCUG motif located at the 3′-UTR paired the Shine-Dalgarno (SD) region at the 5′-UTR. *In vitro* experiments indicated that this interaction promotes mRNA decay and inhibits ribosome loading. Lastly, *in vivo* analysis of bacteria expressing *icaR* mRNA variants demonstrated that deletion or substitution of the UCCCCUG motif strongly decreased the 3′-UTR/Shine-Dalgarno interaction facilitating IcaR expression. As a consequence, PIA-PNAG synthesis and biofilm formation was impaired demonstrating the biological relevance of the 5′-3′-UTRs interaction. This study illustrates that bacterial 3′-UTRs can provide potential strategies for post-transcriptional regulation through an interaction with the SD region at the 5′-UTR. In this case, it is worth noting that base pairing is occurring between the 3′-UTR and the 5′-UTR encoded in the same mRNA.

## Results

### Identification of long 3′-UTRs in the *S. aureus* transcriptome

**Figure 1:**
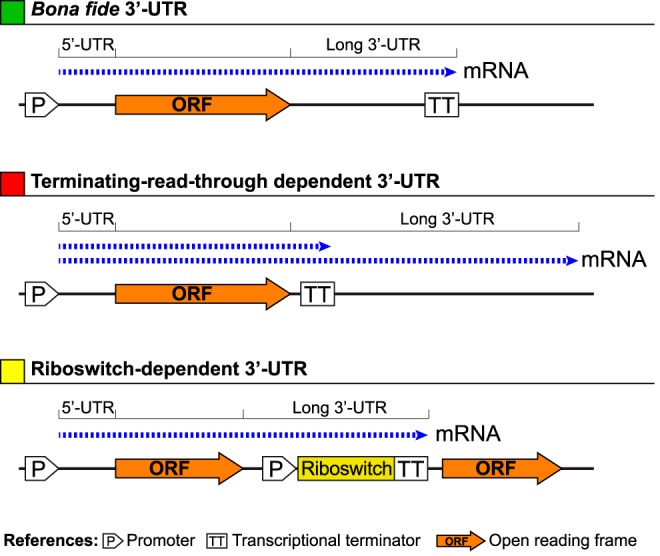
Schematic representation of how long bacterial 3′-UTRs can be generated. A transcript ending in a transcriptional terminator located far away from the corresponding protein stop codon generates a *bona fide* long 3′-UTR. In other cases, despite the presence of a transcriptional terminator (TT) close to the end of the protein stop codon, transcription may continue downstream the predicted TT, generating a terminating-read-through-dependent long 3′-UTR. In addition, several transcripts end at a TT that is part of the expression platform of a riboswitch. In this case the long 3′-UTRs will be generated only when the riboswitch is in an OFF configuration. Otherwise, if the riboswitch is in an ON configuration, a polycistronic transcript is generated.

Finding the 5′ boundaries of mRNAs is a critical step for transcriptional promoter recognition. Thus, in general, bacterial transcriptome analyses have been focused on the identification of mRNA 5′-ends while mRNA 3′-ends mapping has been mostly disregarded, limiting our knowledge about the molecular features inside this mRNA region. To overcome this limitation, we have examined genome wide the 3′ boundaries of the *S. aureus* transcriptome by combining RNA-seq data obtained in a previous study [22] with tiling array hybridization data of four genetically unrelated *S. aureus* strains. The normalized tiling arrays signals and the mapped reads were integrated in a web repository that enables the visualization of the transcriptome information (*Staphylococcus aureus* Transcriptome Browser, http://staph.unavarra.es/). We calculated the 3′-UTR length of each mRNA as the distance between the annotated translational stop codon of the corresponding ORF and the last position of RNA reads downstream. Only 3′-UTRs present in the four strains analysed by tiling were considered in the analysis and, whenever possible, the position of the predicted intrinsic Rho-independent transcriptional terminator was calculated according to the TransTermHP v2.07 program [32]. As a result, we identified 1055 mRNAs carrying *bona fide* 3′-UTRs, that is, transcripts ending at an intrinsic TT (Figure 1 and Figure S1). Remarkably, 34.8% of these mRNAs contained 3′-UTRs longer than 100 nt. Also, we found that transcription of about one third of the mRNAs carrying *bona fide* 3′-UTRs may continue downstream the predicted TTs, thus generating a long terminating-read-through-dependent 3′-UTR (Figure 1 and Figure S1). These 3′-UTRs showed the longest size and usually overlapped with the mRNAs encoded at the opposite DNA strand. This antisense regulation is subsequently followed by RNA degradation induced by the endoribonuclease III (RNase III) [22]. Confirming previous findings in *L. monocytogenes* [20], we also found 24 riboswitch-dependent 3′-UTRs, all of them presenting a length higher than 100 nt (Figure 1 and Figure S1,). In this type of 3′-UTRs, the TT generated when the riboswitch is in an OFF conformation also acts as the TT of the gene encoded upstream of the riboswitch. As a consequence, the 3′-UTR includes the riboswitch sequence. Taken together, these findings indicate that the presence of long 3′-UTRs is very frequent in the *S. aureus* transcriptome and might generate diverse regulatory mechanisms.

### The *icaR* mRNA contains a highly conserved long 3′-UTR

**Figure 2:**
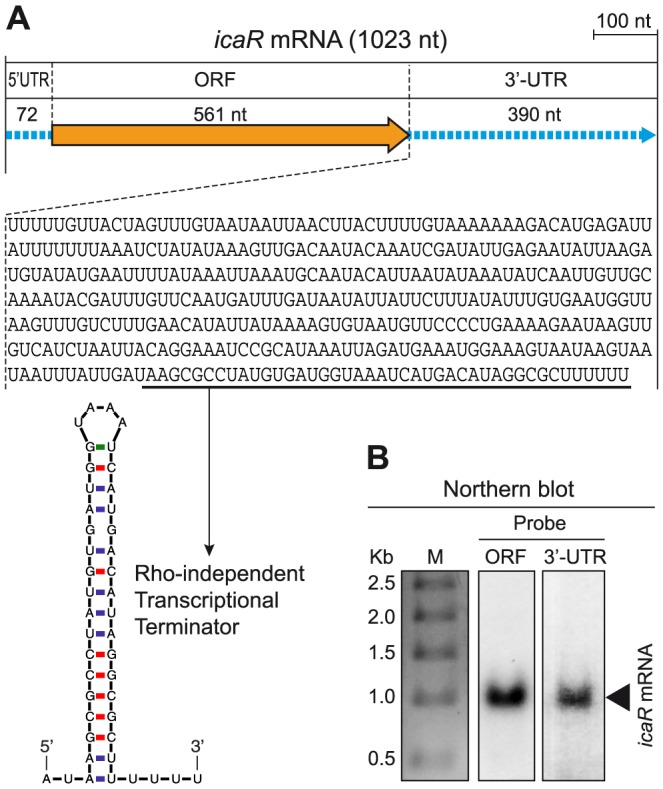
*icaR* mRNA contains a conserved long 3′-UTR. (A) Schematic representation of the *icaR* mRNA molecule, mapped by RACE, showing the length of the 5′-UTR, ORF and 3′-UTR. The nucleotide sequence of the 3′-UTR is shown. The sequence of the transcriptional terminator is underlined and its secondary structure, predicted by Mfold, is shown. (B) Northern blots carried out with two different riboprobes, one targeting the *icaR* ORF region and the other, the *icaR* mRNA 3′-UTR.

To evaluate whether long 3′-UTRs have a functional role in *S. aureus*, we focused on *bona fide* long 3′-UTRs and exclusively concentrated on non-overlapping long 3′-UTRs in order to avoid possible effects due to antisense regulation. Due to the relevance of biofilm formation during *S. aureus* infection, we chose the *bona fide* long 3′-UTR of *icaR* transcript (Figure S2), that encodes a repressor of biofilm formation, as a model to examine whether long 3′-UTRs play a role in controlling the fate of the mRNA and/or its translation process. We first validated the *icaR* mRNA boundaries using a specialized mRACE protocol that uses circularized RNAs [20]. mRACE experiments located *icaR* mRNA 5′ transcriptional start site (TSS) 72-nt upstream from the start codon and the transcriptional termination site (TTS) 390-nt after the stop codon, immediately downstream of the predicted Rho-independent transcriptional terminator (Figure 2A). Northern blot analysis using either a probe complementary to the 3′-UTR region or to the coding region, confirmed the presence of a band of ∼1 Kb that is consistent with the 1023-nt mRNA molecule mapped by mRACE (Figure 2B). We next excluded that the 3′-UTR codes for a peptide using Glimmer v3.02 at the NCBI web page. From these experiments we concluded that *icaR* mRNA contains a long *bona fide* 3′-UTR that accounts for 38% of the complete mRNA molecule (Figure 2A).

Sequence conservation between intergenic regions of different bacterial species has been traditionally used to identify regulatory non-coding RNAs [33]. Indeed, non-coding regions are more permissive to nucleotide substitutions than protein-coding regions, unless the sequence of the “non-coding” region plays a functional role. We compared sequence conservation of the coding sequence and also of the 3′-UTR inside *icaR* mRNA in the 173 *S. aureus* genomes available at the Microbes database from NCBI. Nucleotide variation analysis showed that 19 nt out of the 561 nt corresponding to *icaR* ORF were variable (3.4%). In the case of the 390 nt 3′-UTR, an accumulation of 26 nt changes occurred, which accounts for a 6.6% of this region. In contrast, the region comprised between the TTS of *icaR* mRNA and the TSS of the neighbour *capA* gene transcript showed a variation of 18.8% (Figure S3A). A similar analysis was carried out with the RNAIII molecule, which is a multifunctional regulatory RNA that encodes δ-hemolysin, and that also contains a long 3′-UTR of 352 nt acting as an antisense RNA to repress the translation of target mRNAs [34]. Results again showed a very little nt variation inside both the coding region and the 3′-UTR when compared to the number of changes occurring downstream RNAIII (Figure S3B). Overall, the high degree of conservation present in the 3′-UTR of *icaR* transcript strengthened our hypothesis that this region might play a functional role in *S. aureus*.

### 3′-UTR *icaR* post-transcriptionally modulates IcaR expression

**Figure 3:**
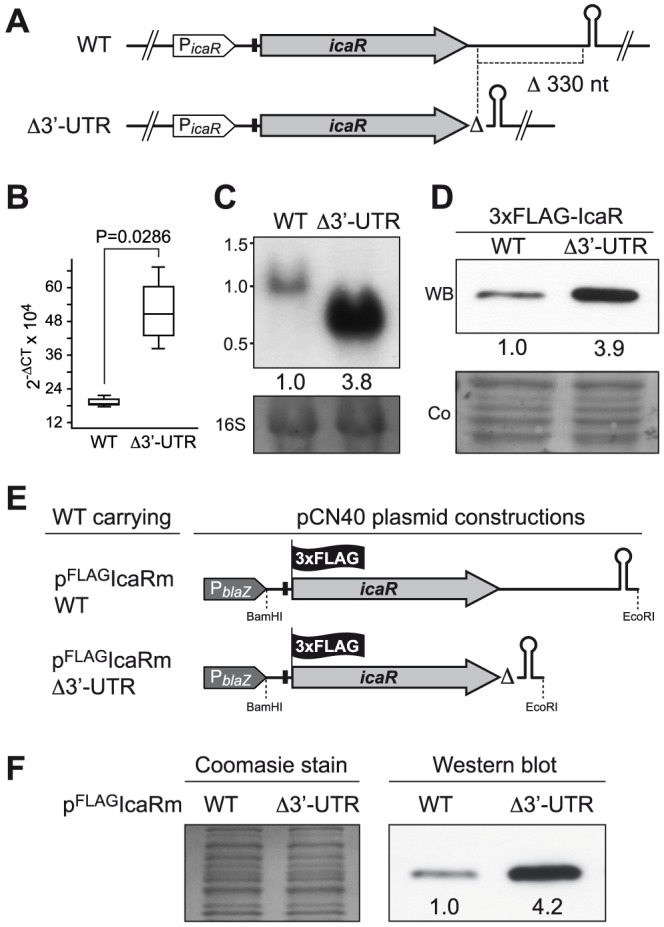
*icaR* 3′-UTR post-transcriptionally modulates IcaR expression. (A) Schematic representation of chromosomal 3′-UTR deletion. Note that the transcriptional terminator is not affected by the deletion. (B) qRT-PCR analysis of *icaR* mRNA levels in *S. aureus* 15981 wild type and Δ3′-UTR strains grown in TSB-gluc at 37°C until exponential phase (OD_600 nm_ = 0.8). The *gyrB* transcript was used as an endogenous control, and the results were expressed as the n-fold difference relative to the control gene (2^−ΔCt^, where ΔCt represents the difference in threshold cycle between the target and control genes). (C) A representative Northern blot showing *icaR* mRNA of wild type and Δ3′UTR strains grown in TSB-gluc at 37°C until exponential phase (OD_600 nm_ = 0.8). Lower panel shows 16S ribosome band stained with ethidium bromide as loading control. (D) A representative Western blot showing IcaR protein levels expressed from strains shown in panel A. The 3XFLAG tagged IcaR protein was detected with commercial anti-3XFLAG antibodies. Numbers below the image show relative band quantification according to densitometry analysis performed with ImageJ (http://rsbweb.nih.gov/ij/). A Coomassie stained gel portion is shown as loading control. (E) Schematic representation of plasmid constructions constitutively expressing the 3XFLAG tagged IcaR protein from the whole *icaR* mRNA or the mRNA carrying the 3′-UTR deletion. (F) A representative Western blot showing IcaR protein levels of strains shown in panel E. The 3XFLAG tagged IcaR protein was detected with commercial anti-3XFLAG antibodies. Densitometry analysis is also shown. On the left, a Coomassie stained gel portion is shown as loading control.

We then sought to determine whether the long 3′-UTR of *icaR* mRNA modulates the expression of the IcaR protein. We generated an isogenic mutant carrying a 330-bp chromosomal deletion that removes most of the 3′-UTR of *icaR* but preserves the intrinsic TT integrity in the *S. aureus* 15981 strain (Figure 3A). We then measured *icaR* mRNA levels of wild type and mutant cells grown until exponential phase (OD_600 nm_ = 0.8) by qRT-PCR. Results showed that deletion of the 3′-UTR induced a ∼3-fold increase in *icaR* mRNA levels (P = 0.0286) (Figure 3B). This increase was also observed by Northern blot (Figure 3C). Note that the presence of a band corresponding to the expected ∼0,7 kb in the Northern blot implies that the deletion of the 3′-UTR did not affect transcription termination of the mutated *icaR* mRNA (Figure 3C). Next, we asked whether the increase in the amount of *icaR* mRNA correlated with higher levels of IcaR protein. We tagged the chromosomal copy of *icaR* gene with the 3XFLAG sequence in both the wild type and Δ3′-UTR mutant strains. Results showed that Δ3′-UTR mutant strain produced significantly higher levels of IcaR protein compared to the wild type strain (Figure 3D).

The increase in the amount of *icaR* mRNA/IcaR protein in the absence of the 3′-UTR could be explained either by the existence of higher transcriptional rates from the *icaR* promoter or by an increased stability of *icaR* mRNA or even by a combination of both processes. To distinguish between these possibilities, we uncoupled transcriptional and post-transcriptional regulation by ectopically expressing either the entire *icaR* mRNA or the *icaR* mRNA lacking the 3′-UTR under the control of a constitutive promoter. In both cases, IcaR was tagged with a 3XFLAG epitope at the N-terminal generating plasmids p^FLAG^IcaRm_WT and p^FLAG^IcaRmΔ3′-UTR (Figure 3E). Western blot analysis revealed that IcaR protein was produced in higher levels (∼4 fold) in the strain harbouring p^FLAG^IcaRmΔ3′-UTR compared to the strain harbouring p^FLAG^IcaRm_WT (Figure 3F). This result suggests that the 3′-UTR mediated regulation occurs at the post-transcriptional level.

**Figure 4:**
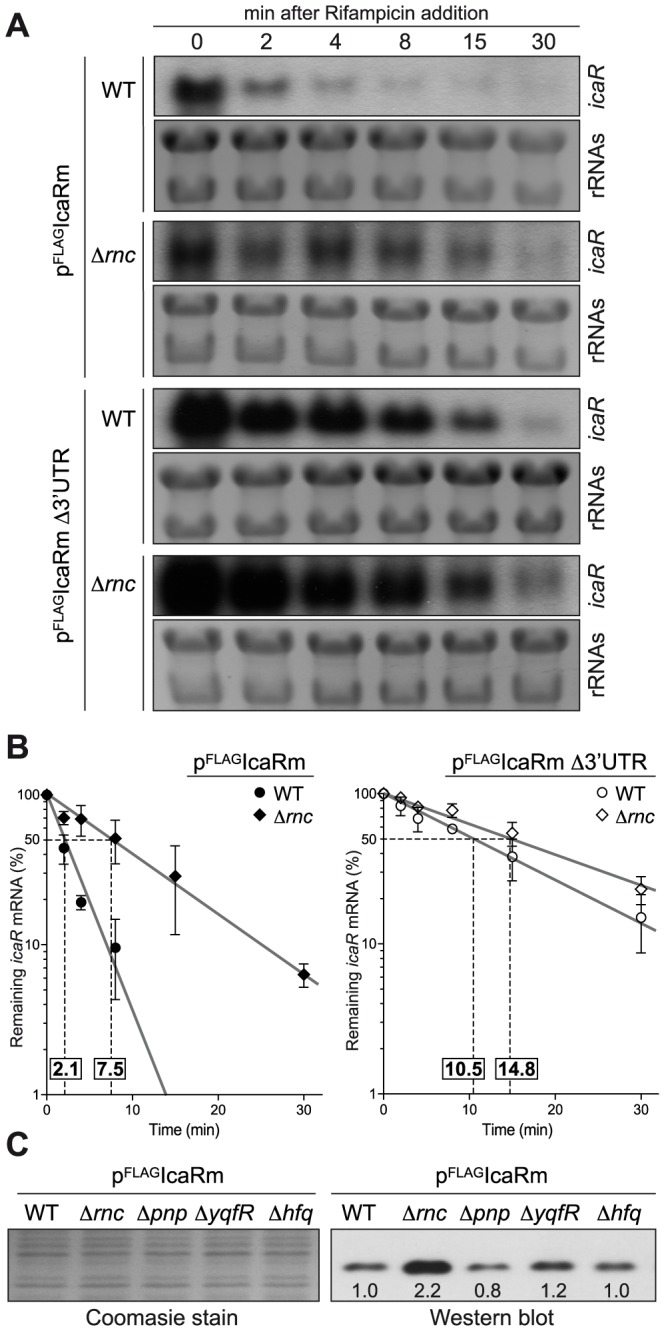
Deletion of *rnc* gene, which encodes the double stranded endoribonuclease RNase III, affects *icaR* mRNA stability and IcaR protein levels. (A) Half-life measurement of *icaR* wild type and Δ3′-UTR mRNAs constitutively expressed in the wild type and Δ*rnc* mutant strains. These strains were grown in TSB-gluc at 37°C until exponential phase (OD_600 nm_ = 0.8) and then rifampicin (300 µg/ml) was added. Samples for RNA extraction were taken at the indicated time points (min). The experiment was repeated three times and representative images are shown. (B) Levels of *icaR* mRNA were quantified by densitometry of Northern blot autoradiographies using ImageJ (http://rsbweb.nih.gov/ij/). Each of mRNA levels was relativized to mRNA levels at time 0. The logarithm values of relative mRNA levels were subjected to linear regression analysis and plotted as a function of time. Error bars indicate the standard deviation of mRNA levels from three independent experiments. Dashed lines indicate the time at which 50% of mRNA remained. The half-life of mRNAs is shown above of X-axis. (C) Representative Western blot showing IcaR protein levels in different mutant strains constitutively expressing the 3XFLAG tagged IcaR from the P*blaZ* promoter. Tagged IcaR protein was detected with commercial anti-3XFLAG antibodies. On the left, a Coomassie stained gel portion is shown as loading control. *rnc*, double-stranded endoribonuclease RNase III; *pnp*, polynucleotide phosphorylase PNPase; *yqfR*, (SAOUHSC_01659), ATP-dependent RNA helicase containing a DEAD box domain; *hfq*, RNA chaperone, host factor-1 protein.

We then explored the possibility that the 3′-UTR may reduce mRNA stability. A comparison of *icaR* mRNA stability revealed that *icaR* mRNA half-life increased from 2.1 min in the wild type to more than 10 min in the Δ3′-UTR strain (Figure 4A and B). Degradation of mRNA can follow several pathways involving a combination of exo- and endoribonucleases and RNA-binding proteins [17], [18]. In order to determine the proteins involved in *icaR* mRNA decay, we compared the relative abundance of IcaR protein in the wild type 15981 *S. aureus* strain and isogenic deletion mutants in proteins that might affect mRNA stability such as PNPase, RNase III, RNA helicases and Hfq. Our reasoning was that the IcaR protein should accumulate in the mutants coding for proteins that might be required for *icaR* mRNA decay. The results revealed that only deletion of *rnc*, which encodes RNase III, an endoribonuclease able to degrade double stranded RNA, caused a significant increase in IcaR levels (Figure 4C). Accordingly, *icaR* mRNA half-life increased from 2.1 min in the wild type to 7.5 min in the *rnc* mutant (Figure 4A and B). Taken together, these results provide compelling evidence that the 3′-UTR is involved in promoting *icaR* mRNA decay in a process that is dependent, at least in part, on RNase III activity.

### Base pairing interaction between 3′- and 5′-UTR of *icaR* mRNA

RNA secondary structures play key roles in post-transcriptional regulatory mechanisms including RNA decay [18], recruitment of RNA-binding proteins [11] and riboswitches [15], [16]. To gain insight into the 3′-UTR mediated *icaR* mRNA decay, the secondary structure of *icaR* mRNA was predicted using the Mfold program (http://mfold.rit.albany.edu/) [35]. Surprisingly, the prediction showing the lowest energy (initial DG = −230.50 Kcal mol-1) revealed a pairing between the 3′-UTR region (from 890 to 982-nt) and the 5′-UTR SD region (from 4 to 67-nt) of *icaR* mRNA (Figure S4). The predicted 5′-3′-UTR interacting region comprised around 40 base-paired nucleotides (including G-U interactions).

Because the interaction between the 3′-UTR and the 5′-UTR can be formed either in *cis* or *trans*, we analysed if the full-length *icaR* mRNA was able to fold into various conformations *in vitro*. As expected, the mRNA renatured in TE buffer migrated as a single band on an agarose gel. In contrast, the *icaR* mRNA which was renatured at 37°C in a buffer containing KCl and MgCl_2_, presented two distinct bands (Figure S5). The slower migrating band corresponded to a molecular mass consistent with a dimeric form of *icaR* mRNA (Figure S5).

**Figure 5:**
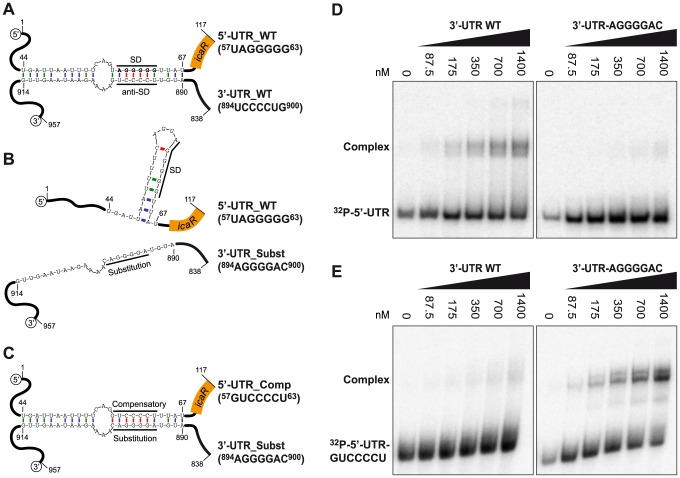
A UCCCCUG motif is necessary for the interaction between the 3′-UTR and the Shine-Dalgarno region of *icaR* mRNA *in vitro*. (A) Schematic representation of the 5′-3′-UTRs interaction. A UCCCCUG motif located at the 3′-UTR pairs the UAGGGGG Shine-Dalgarno region located at the 5′-UTR. The numbers indicate the relative position of the nucleotides in the full-length *icaR* mRNA (B) Substitution of the ^894^ UCCCCUG ^900^ motif by ^894^ AGGGGAC ^900^, disrupts the pairing predicted by Mfold program. (C) Introduction of a compensatory mutation sequence (^57^ GUCCCCU ^63^) in the 5′-UTR, complementary to the substituted ^894^ AGGGGAC ^900^ motif, restores complex formation. (D) Gel shift analysis of the 5′ and 3′-UTR *icaR* mRNA interaction. The ^32^P-labeled 5′-UTR fragment (1–117-nt) was incubated with increasing concentrations of unlabeled 3′-UTR (3′-UTR WT) or substituted 3′-UTR (3′-UTR-AGGGGAC) (838–957-nt). (E) Similarly, the ^32^P-labeled compensatory-5′-UTR fragment (^32^P-5′-UTR-GUCCCCU) was incubated with increasing concentrations of unlabeled 3′-UTR (3′-UTR WT) or substituted 3′-UTR (3′-UTR-AGGGGAC). After 30 min of incubation at 37°C, the mixture was analysed by electrophoresis in a native 5% polyacrylamide gel and PhosphorImager (Molecular Dynamics).

A more detailed analysis of the predicted secondary structure revealed that the pairing region includes a ^894^ UCCCCUG ^900^ motif located 260-nt downstream of the IcaR stop codon complementary to the SD region (^57^ UAGGGGG ^63^) (Figure 5A and S4). This UCCCC sequence motif has been previously described in several sRNAs of *S. aureus* where it promotes fast binding to target mRNAs and prevents the formation of the ribosomal initiation complex [36], [37]. To experimentally monitor the interaction between the ^57^ UAGGGGG ^63^ and ^894^ UCCCCUG ^900^ motifs, we performed RNA gel shift assays using a ^32^P-labelled 5′-UTR fragment (117-nt) including the SD sequence and increasing concentrations of a 3′-UTR fragment (120-nt) containing either the wild type ^894^ UCCCCUG ^900^ or a substituted ^894^ AGGGGAC ^900^ motif, which disrupts sequence pairing predicted by Mfold program (Figure 5B). The data showed that the 3′-UTR fragment binds to the 5′-UTR fragment carrying the SD with rather low affinity binding (>700 nM). However this interaction is specific because substitution of the ^894^ UCCCCUG ^900^ motif by ^894^ AGGGGAC ^900^ severely decreased binding with the 5′-UTR fragment (Figure 5D). Introduction of a compensatory mutation (^57^ GUCCCCU ^63^) in the SD sequence complementary to the substituted ^894^ AGGGGAC ^900^ motif restored complex formation (Figure 5C and 5E). Together, these data show that the *icaR* mRNA 3′-UTR specifically anneals to the 5′-UTR in a region that overlaps with the ribosome-binding site.

### The 5′-3′-UTRs pairing provides a substrate for RNase III cleavage

**Figure 6:**
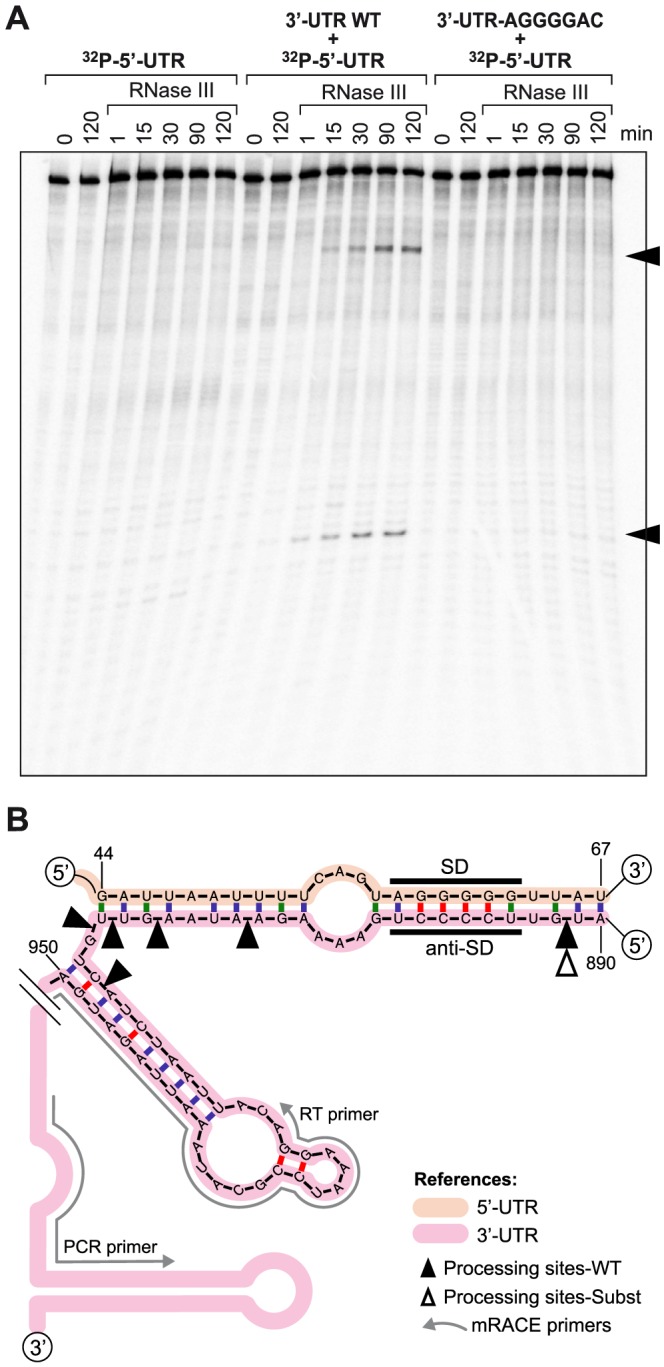
*In vitro* and *in vivo* RNase III-mediated processing of the double stranded region generated by *icaR* 5′-3′-UTRs interaction. (A) *In vitro* RNase III activity assay. A ^32^P-labelled 5′-UTR fragment was incubated with purified recombinant *S. aureus* RNase III during different times in the absence or presence of either the 3′-UTR fragment or the substituted 3′-UTR fragment. The two RNA bands that are generated by the presence of the wild type 3′-UTR are indicated with arrows. (B) Schematic representation showing *in vivo* mRACE results. Mapping of *icaR* mRNA fragments naturally generated *in vivo* was carried out with circularized RNAs and two outward primers (RT and PCR) that pair next to the transcriptional terminator. Black and white triangles indicate *in vivo* processing sites identified in the *icaR* mRNA wild type and the *icaR* mRNA with the UCCCC substitution respectively.

Because pairing between the 3′- and 5′-UTRs creates a double stranded region, we sought to determine whether RNase III was capable of cleaving *icaR* mRNA at the pairing region. We performed *in vitro* cleavage assays using a uniformly ^32^P-labelled 5′-UTR fragment mixed with the 3′-UTR fragment in the presence of the purified recombinant *S. aureus* RNase III. Results showed that RNase III is able to cleave the 5′-UTR fragment, only in the presence of the 3′-UTR, generating two bands. Thus, we could not detect processed bands either in the absence or in the presence of a 3′-UTR with the AGGGGAC substituted motif (Figure 6A).

To test whether our *in vitro* assay mimicked RNase III capacity to cleave the 5′-3′-UTR duplex of *icaR* mRNA *in vivo*, we performed mRACE analysis with circularized RNA from extracts purified from the wild type, the 3′-UTR *icaR*-SUBST and *rnc* mutant strains. Results showed several processing sites located at the double stranded region of the UCCCC motif when wild type RNA was used (Figure 6B). In contrast, we were able to detect only one processing site at the UCCCC region in the 3′-UTR *icaR*-SUBST strain. In agreement with the *in vitro* results, no processing sites could be detected in the assay performed with the RNA extract purified from the Δ*rnc* mutant. These results are consistent with the conclusion that RNase III directs the processing of a double stranded region formed by the pairing between *icaR* 3′-UTR and 5′-UTR both *in vitro* and *in vivo*.

### Interaction between *icaR* 3′-UTR and SD region prevents the formation of the translational initiation complex

**Figure 7:**
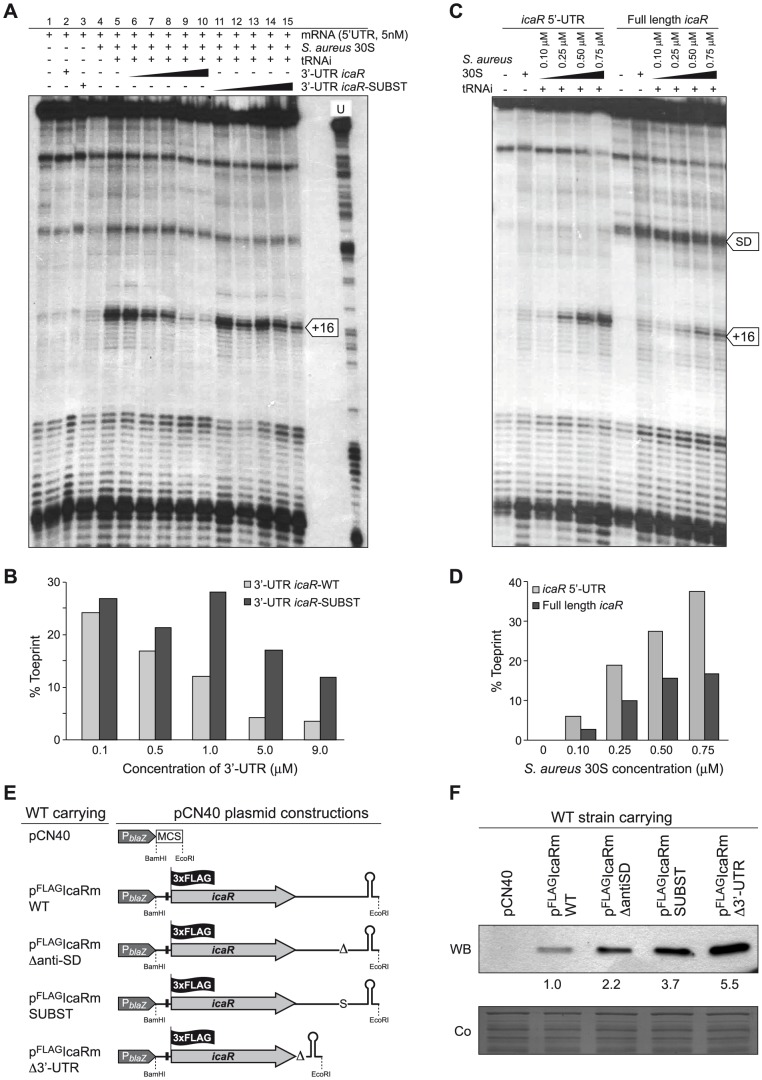
The 5′-3′-UTRs interaction interferes with the translational initiation complex. (A, B) Formation of the ternary complex between *icaR* 5′-UTR fragment (5 nM), *S. aureus* 30S ribosomal subunit, and initiator tRNA was monitored in the absence or in the presence of increasing concentrations of wild-type 3′-UTR fragment and substituted 3′-UTR fragment. The toeprint at position +16 is indicated. The quantification of the toeprint (B) was first normalized according to the full-length extension product bands using the SAFA software [79], and the toeprint signal (given in %) represents the yield of the toeprint obtained in the presence of the competitor RNA versus the yield of the toeprint obtained in the absence of the competitor RNA. (C, D) Formation of the ternary complex with the 5′-UTR fragment and the full-length *icaR* mRNA molecule was monitored using different *S. aureus* 30S concentrations. A reverse transcriptase pause at the Shine-Dalgarno (SD) sequence occurring in the full-length *icaR* mRNA molecule is indicated with an arrow. The quantification of toeprint experiment (D) is described above in B. (E) Schematic representation of plasmid constructions constitutively expressing the 3XFLAG tagged IcaR protein from the different *icaR* mRNA alleles. (F) A representative Western blot showing IcaR protein levels in strains shown in panel E. The 3XFLAG tagged IcaR protein was detected with commercial anti-3XFLAG antibodies. Band quantification according to densitometry analysis is shown. A Coomassie stained gel portion is shown as loading control.

Because the interaction of the 3′-UTR with the 5′-UTR of *icaR* mRNA coincides with the ribosome binding site (RBS), we expected that the 3′-UTR should prevent ribosome loading on the *icaR* mRNA. Toeprint assays were performed to analyze the formation of the ternary ribosomal initiation complexes including purified *S. aureus* 30S ribosomes, initiator tRNA^Met^ and various fragments of *icaR* mRNA. The experiment was first done on a truncated version of *icaR* mRNA containing the whole 5′-UTR and 75 nts from the coding sequence (Figure 7A). As expected, the formation of the ternary complex was able to block the elongation of a cDNA primer by reverse transcriptase (RT) to produce a toeprint signal at 16 nt downstream of the initiation codon (Figure 7A). The addition of increasing concentrations of the 3′-UTR significantly reduced ribosome loading onto the *icaR* 5′-UTR in a concentration-dependent manner. In contrast, increasing amounts of the mutated 3′-UTR *icaR*-SUBST, that cannot form a complex with the *icaR* 5′-UTR (Figure 5), did not prevent ribosome loading onto the mRNA (Figure 7A and 7B). We then compared the ability of the *S. aureus* 30S to recognize the 5′-UTR and the whole *icaR* mRNA (Figure 7C). Quantification of the data showed that the 5′-UTR fragment of *icaR* is recognized by the 30S more efficiently than the full-length mRNA (Figure 7D). In addition, a RT pause at the SD sequence was slightly stronger with the full-length mRNA than with the 5′-UTR, probably due to the interaction of the 3′-UTR with the SD sequence (Figure 7C). We also performed toeprinting assays on *S. aureus spa* mRNA which carries a short 5′-UTR, an unstructured ribosome binding site, and a similar SD and initiation codon as found in *icaR* mRNA (Figure S6A). The data showed that a large proportion of *spa* mRNA was able to form an active initiation complex, and that the 30S recognized *spa* mRNA better than the 5′-UTR and the full-length *icaR* mRNA (Figure S6B and C). All in all, these results indicate that pairing between the 3′-UTR and the SD region specifically hinders ribosome binding to the *icaR* transcript and that the 5′-UTR is weakly recognized by *S. aureus* 30S.

To demonstrate that the ^894^ UCCCCUG ^900^ motif is able to regulate IcaR synthesis *in vivo*, we evaluated IcaR protein levels of wild type and RNase III mutant strains harbouring plasmids that constitutively expressed *icaR* mRNA derivatives with a deletion of the ^894^ UCCCCUG ^900^ motif (p^FLAG^IcaRm Δanti-SD) or carrying the substitution of this motif by ^894^ AGGGGAC ^900^ (p^FLAG^IcaRm SUBST) (Figure 7E). Strains expressing *icaR* mRNA derivatives with a deletion or substitution of the UCCCCUG motif accumulated higher levels of IcaR protein, compared to the strain producing wild type *icaR* transcript (Figure 7F). Interestingly, accumulation of IcaR protein was the highest in the Δ*rnc* mutant strain expressing the *icaR* mRNA with the UCCCCUG substitution (Figure S7). These results strongly suggest that both processes, inhibition of ribosome loading and cleavage by RNase III, modulate IcaR levels.

### Biological relevance of the SD/UCCCC base pairing

**Figure 8:**
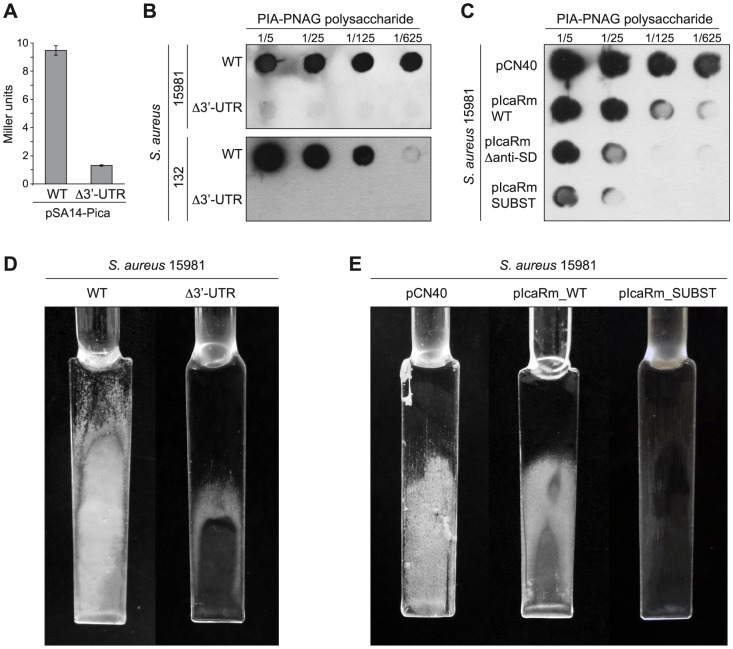
*In vivo* relevance of the interaction between the 3′-UTR and the Shine-Dalgarno region of *icaR* mRNA. (A) β-galatosidase assays measuring *icaA* promoter activity in the wild type and Δ3′-UTR strains grown in TSB-gluc at 37°C until exponential phase (OD_600 nm_ = 0.8). Each bar represents the average of three independent assays. (B) Consequences of *icaR* mRNA 3′-UTR deletion on PIA-PNAG exopolysaccharide synthesis and biofilm production of *S. aureus* 15981 and 132 strains. (C) *In vivo* effects of either the mutation or the substitution of the ^894^ UCCCCUG ^900^ motif on PIA-PNAG synthesis. Quantification of PIA-PNAG exopolysaccharide biosynthesis by dot-blot. Serial dilutions (1/5) of the samples were spotted onto nitrocellulose membranes and PIA-PNAG production was detected with specific anti-PIA-PNAG antibodies. (D) Biofilm development of the wild type and Δ3′-UTR strains grown in microfermentors under continuous flow for 8 h at 37°C. The glass slides where bacteria form the biofilm are shown. (E) Biofilm development of the *S. aureus* 15981 with the pCN40, pIcaRm_WT and pIcaRm_SUBST plasmids grown in microfermentors under continuous flow for 8 h at 37°C.

IcaR represents the checkpoint of *S. aureus* biofilm formation since it binds to a 42-bp region located just upstream of the *icaA* gene to directly inhibit *icaADBC* transcription [31]. To examine the biological relevance of the *icaR* mRNA 3′-UTR in controlling *in vivo* multicellular behaviour, we first checked if the high IcaR levels observed in the Δ3′-UTR mutant strain were able to affect *icaADBC* operon transcription. For that, we compared *icaADBC* promoter activity in the wild type *icaR* strain and its corresponding Δ3′-UTR mutant using a transcriptional reporter plasmid comprising the *lacZ* gene fused to the *ica* promoter (P*ica*). As expected, β-galactosidase assays revealed that the activity of the P*ica* promoter was ∼7-fold lower in the Δ3′-UTR mutant than in the wild type strain (Figure 8A). Then, we determined the effect of the 3′-UTR deletion on the capacity of two genetically unrelated *S. aureus* strains (*S. aureus* 15981 and *S. aureus* 132) to synthesize PIA-PNAG exopolysaccharide and develop a biofilm. Dot-blot assays using anti PIA-PNAG specific antibodies showed that the synthesis of PIA-PNAG was completely inhibited in both Δ3′-UTR mutant strains (Figure 8B). Accordingly, Δ3′-UTR mutant strains lost the capacity to develop a biofilm under continuous-flow conditions in microfermenters (Figure 8D). Then, to assess specifically the relevance of ^894^ UCCCCUG ^900^ motif for the regulation of PIA-PNAG synthesis and biofilm development, we tested PIA-PNAG levels and biofilm formation capacity in *S. aureus* 15981 wild type strain transformed with plasmids carrying either full length *icaR* mRNA (pIcaRm_WT) or derivatives with deletion or substitution of the UCCCCUG motif (pIcaRm_Δanti-SD and pIcaRm_SUBST respectively). Strains producing *icaR* mRNA derivatives accumulated lower levels of PIA/PNAG (Figure 8C), and displayed a significant reduction in the capacity to produce a biofilm compared to the strain expressing full length *icaR* mRNA (Figure 8E). These experiments demonstrate the biological relevance of the *icaR* 3′-UTR and the UCCCCUG motif in controlling PIA-PNAG production and biofilm development by adjusting IcaR repressor protein levels through a post-transcriptional event involving the interaction of a 3′-UTR and a 5′-UTR of the same mRNA.

## Discussion

### 5′-3′-UTR base pairing to modulate mRNA translation in bacteria

Our genetic and biochemical analysis revealed that *icaR* translation depends on the ability of the long 3′-UTR of *icaR* to interact with a 40 nt complementary region of the 5′-UTR. Thus, inhibition of this interaction by either deleting or substituting few complementary nucleotides in the 3′-UTR causes the accumulation of IcaR protein *in vivo*. Because both 5′- and 3′-interacting motifs are encoded in the same mRNA molecule, the question arises as to whether the interaction occurs intramolecularly (3′- and 5′-UTRs of the same mRNA molecule) or intermolecularly (3′- and 5′-UTRs of different mRNA molecules). With respect to the first possibility, that is circularization of the mRNA, meaning the formation of a physical bridging of 5′- and 3′-ends, it is a widely accepted mechanism of translational regulation in eukaryotes and virus. Proteins associated with the 5′-cap- and 3′-poly(A) tail are usually required to mediate UTRs interaction and depending on the proteins involved, the interaction can stimulate or repress mRNA translation [1], [38], [39]. Transcript circularization can also be initiated by simpler RNA interactions in some viruses. For example, during the translation process of several RNA viruses such as *Barley yellow dwarf luteovirus* and dengue virus, the positive-strand RNA genome forms a closed loop by direct base-paring between complementary regions located at the 3′-UTR and the 5′-UTR to confer translation initiation at the 5′-proximal AUG [40], 41. Transposition of this scenario to the 5′-3′-UTRs interaction described here needs to reconcile the widely established concept that transcription and translation processes are coupled in bacteria. If ribosomes started translation of *icaR* mRNA before the RNA polymerase synthetized the UCCCC motif, the 3′-UTR would not be able to modulate the initial rounds of *icaR* translation. Comparative toeprinting assays showed that the 5′-UTR of *icaR* is recognized by the ribosome less well than *spa* mRNA which is characterized by an unstructured RBS. This data suggested that the 5′-UTR would adopt a structural fold to impair efficient 30S binding during transcription but sufficiently unstable to be displaced by the 3′-UTR. In such model, the 3′-end of *icaR* would trigger a refolding of the 5′-UTR to promote the access of RNase III and to fully impair ribosome loading. Such a step-wise mechanism would be reminiscent to the temporal translational control of *E. coli hok* mRNA involved in programmed cell death [42]. We cannot exclude that translation initiation might be delayed by an unknown mechanism, i.e. involvement of trans-acting factors, while *icaR* mRNA is fully transcribed.

Alternatively, intermolecular interactions between identical RNA molecules through complementary sequences have been shown to be essential for retroviral RNAs [43], formation of ribonucleoprotein particles for transport and localization of *bcd* mRNA (bicoid) during *Drosophila* development [44], [45] and the formation of the cyclic hexamer pRNA needed for efficient *in vitro* packaging of the *Bacillus subtilis* bacteriophage phi29 genome [46], [47]. In these examples, the intermolecular interaction provides a mechanism to recruit the mRNA molecules in specific structural complexes.

The finding that *icaR* mRNA, and not the 5′-UTR, can form dimers *in vitro* is consistent with an interaction between the 3′-UTR of one *icaR* mRNA molecule and the 5′-UTR of another *icaR* mRNA molecule in *trans* (Figure S5). However, ectopic expression of either the 3′-UTR or the full-length *icaR* mRNA was unable to modify the expression of the chromosomal copy of IcaR questioning the relevance of the intermolecular interaction *in vivo* (data not shown). Furthermore, results of toeprint and RNase III cleavage assays could be explained considering that the interaction occurs either in *cis* or *trans*. Therefore, more detailed studies examining the structure of *icaR* mRNA and the environmental signals that modulate the 5′-3′-UTRs interaction are required before conclusively establishing the intra- or intermolecular mechanisms by which the 3′-UTR might control *icaR* translation and biofilm formation.

Another question that remains to be addressed is whether 5′-3′-UTRs interaction requires the participation of *trans*-acting factors that could use molecular mimicry to sequester the 3′-UTR away from the 5′-UTR and change the SD region from an open to a closed structure. Although we have shown that neither Hfq nor RNA helicase (YqfR) affect IcaR expression, we cannot exclude that other RNA binding proteins or unknown sRNAs might participate in *icaR* 5′-3′ mRNA interaction in response to environmental signals.

### Biological relevance of 5′-3′-UTRs interaction in bacteria

**Figure 9:**
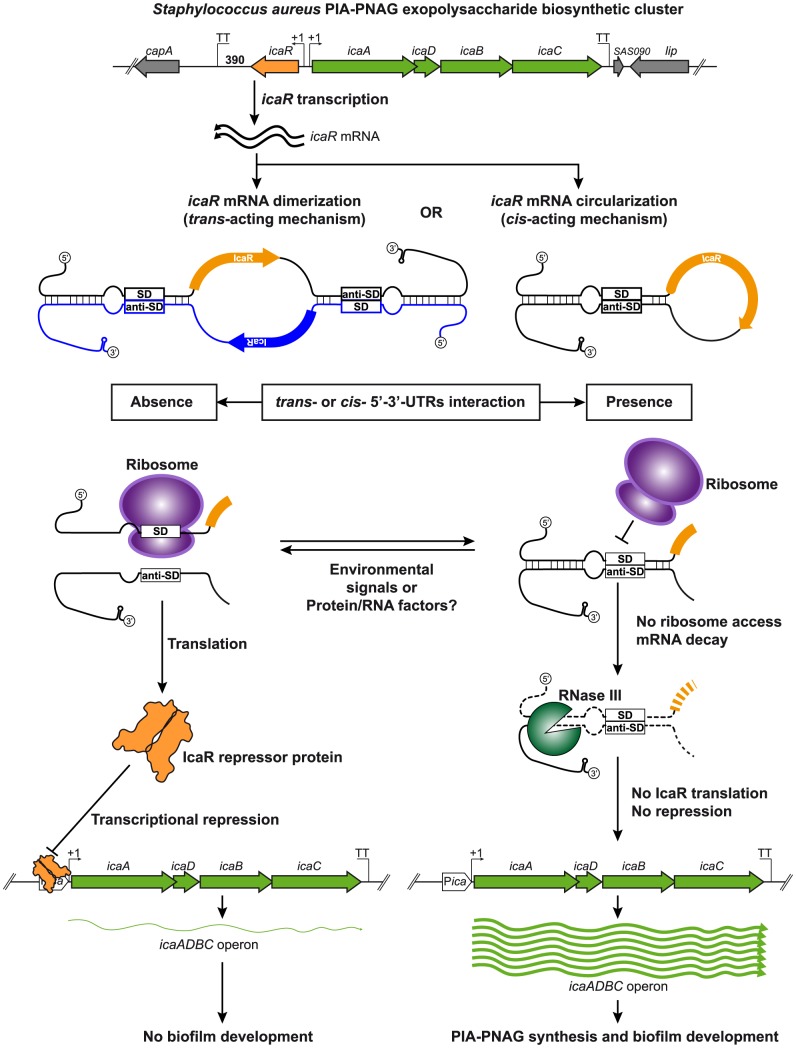
Modulation of IcaR expression by 5′-3′-UTRs interaction. A model of the potential post-transcriptional regulatory mechanism controlling IcaR expression mediated by the 3′-UTR interaction with the Shine-Dalgarno region is shown. Once *icaR* gene is transcribed, the 3′-UTR interacts either in *trans* or *cis* with the 5′-UTR through the anti-SD UCCCCUG motif. This interaction has two main consequences: i) it interferes with ribosome access to the SD region to inhibit the formation of the translational initiation complex and ii) it promotes RNase III-dependent mRNA decay. In consequence, IcaR repressor is less expressed and thus *icaADBC* transcription occurs, favouring PIA-PNAG biosynthesis and biofilm development. When the interaction between *icaR* 3′- and 5′-UTR regions does not happen, ribosome binds the SD and proceeds with IcaR protein translation. The resulting IcaR protein binds to *icaADBC* operon promoter inhibiting its transcription and consequently biofilm formation.

Whatever the detailed interaction mechanisms involved, our results indicated that the ability of the 3′-UTR to interact with the 5′-UTR has profound consequences on the synthesis of IcaR and biofilm development (Figure 9). On one hand, pairing of 3′- and 5′-UTR regions provides a double stranded RNA substrate for RNase III activity, which accelerates *icaR* mRNA decay. On the other hand, 5′-3′-UTRs pairing also hinders the formation of the translational complex. There are examples in the literature in which pairing between 3′- and 5′-UTRs modulates translation in bacteria [48], [49]. As regards the *hok*/*sok* toxin-antitoxin system of plasmid R1, the 3′-end of the full-length *hok* mRNA folds back onto the translational initiation region inhibiting translation [49]. Consequently, the full-length *hok* mRNA was found to be translationally silent whereas a truncated version with a deletion of the 3′-end was active. Novick and co-workers proposed a fold back interaction between the 5′-end and the 3′-end of RNAIII [50]. Although much shorter than the initial computational prediction, this interaction was later confirmed through the analysis of the secondary structure of RNAIII using enzymes and chemical probes [51]. It is worth noting that RNAIII (514 nt), the most studied regulatory RNA in *S. aureus*, is actually an mRNA encoding a small peptide (δ–hemolysin) of 26 amino acids. This implies that the main regulatory region of RNAIII corresponds to a long 3′-UTR of 354-nt that folds in several loops to enable pairing with different target mRNAs to repress their translation [34]. Less known is the capacity of RNAIII to modulate the expression of its own gene, δ–hemolysin [48]. It has been shown that deletion of the 3′-end of RNAIII abolishes a temporal delay between the transcription of RNAIII and its translation. Although the mechanism was not clarified, it was proposed that the 3′-end might fold back to block translation and that a specific cellular factor would be required to unfold the molecule to allow δ–hemolysin translation [48], anticipating the results described in this study.

A common feature between RNAIII and *icaR* mRNA is the presence of UCCCC motifs at the 3′-UTRs [52]. Interestingly, this motif has also been found in several *S. aureus* regulatory sRNAs [37]. In the case of RNAIII and RsaE, the UCCCC motif pairs with mRNA targets in *trans* whereas the UCCCC of *icaR* pairs the SD encoded in the same mRNA molecule. Nevertheless, we cannot exclude that the UCCCC motif of *icaR* 3′-UTR pairs other mRNA targets. Indeed, mRNA target predictions using the *RNA*Predator web server [53] identified several mRNAs whose SD regions might pair with *icaR*-UCCCC region. Interestingly, some of this putative mRNA targets encode proteins that might be involved in biofilm development such as N-acetylglucosaminyl transferase, teichoic acid biosynthesis protein B, spermidine/putrescine ATP binding ABC transporter protein and the iron transcriptional regulator *fur*. Further studies will be needed to elucidate whether the *icaR* UCCCC motif interacts in *trans* with other mRNAs.

### Potential regulatory 3′-UTRs will be broadly distributed in bacteria

Why have bacterial 3′-UTRs gone unnoticed? One possible explanation is that, up to now, bacterial transcriptome analyses have provided very limited knowledge about 3′-UTRs structural features because they have been focused on primary 5′ boundary identification [54]–[61]. Also, the fact that bacterial genomes are very compact and the notion that long 3′-UTRs are restricted to complex organisms [1], [62] has created the feeling that bacterial genomes do not have room to allocate more than few small non-coding RNAs within their short IGRs. However, here, we anticipate that bacterial 3′-UTRs might contain potential regulatory elements contributing to gene expression by different mechanisms, probably including similar ones to the 5′-3′-UTRs interaction described in this study. Our transcriptome analysis revealed that at least 35% of the *S. aureus* mapped mRNAs contained 3′-UTRs longer than 100-nt and that 68% of the mRNAs had a 3′-UTR longer than their corresponding 5′-UTR. These results are in line with another study showing that large UTRs are more frequently found in the 3′- than in the 5′-end in *S. aureus* [63]. Because the average size of the 1,059 intrinsic Rho-independent TTs predicted by TransTermHP in *S. aureus* [32], with a confidence higher than 90%, is 33±10-nt, around 40–50-nt should be a sufficient length to allocate the transcriptional terminator sequence. Therefore, the prevalent high length (>100 nt) is a strong evidence for the potential of 3′-UTRs to allocate putative regulatory elements. Localization of such elements at the 3′-UTR compared with the 5′-UTR of the transcript may present some advantages, since RNA secondary structure in the 3′-UTR is not constrained by the translational process. In this respect, it is interesting to note that the *icaR* mRNA encoded in other staphylococcal species such as *S. epidermidis, S. simiae*, *S. caprae*, and *S. capitis* also carries a long 3′-UTR (365, 482, 369 and 380 nt respectively), though it is not conserved at the sequence level. Interestingly, the UCCCC motif is not present in the *icaR* 3′-UTR of these species. These differences may allow adjusting the biofilm formation process to particular niches in a species-specific manner. Understanding how distinct 3′-UTRs of homologous genes modulate protein expression will be a promising strategy to uncover the regulatory potential of bacterial 3′-UTRs.

## Materials and Methods

### Oligonucleotides, plasmids, bacterial strains and culture conditions

Bacterial strains, plasmids and oligonucleotides used in this study are listed in Table S1, Table S2 and Table S3 respectively. *Staphylococcus aureus* strains were grown in trypticase soy broth supplemented with 0.25% glucose (TSB-gluc) (Pronadisa). *Escherichia coli* was grown in LB broth (Pronadisa). When required for selective growth, medium was supplemented with appropriated antibiotics at the following concentrations: erythromycin (Em), 1.5 µg ml^−1^ and 20 µg ml^−1^; ampicillin (Amp), 100 µg ml^−1^.

### RNA extractions

Bacteria were grown in 20 ml of TSB-gluc at 37°C under shaking conditions (200 rpm) until the culture reached an OD_600 nm_ of 0.8. Cultures were centrifuged, the pellets were frozen in liquid nitrogen and stored at −80°C until needed. Total RNA from bacterial pellets was extracted using the TRIzol reagent method as described [20]. Briefly, bacterial pellets were resuspended into 400 µl of solution A (glucose 10%, Tris 12.5 mM, pH 7.6, EDTA 10 mM) and mixed with 60 µl of 0.5 M EDTA. Resuspended cells were transferred into Lysing Matrix B tubes (MP Biomedicals) containing 500 µl of acid phenol pH 4.5 (Ambion) and mixed. Bacteria were mechanically lysed with a Fastprep apparatus (BIO101) at speed 6.0 during 45 s at 4°C. After lysis, tubes were centrifuged for 10 min at 17,900 g at 4°C. The aqueous phase was transferred to 2-ml tubes containing 1 ml of TRIzol (Invitrogen), mixed, and incubated for 5 min at room temperature. 100 µl of chloroform were added, mixed gently, and incubated for 3 min at room temperature. Tubes were centrifuged for 10 min at 17,900 g at 4°C. The aqueous phase was transferred into a 2-mL tube containing 200 µl of chloroform, mixed, and incubated for 5 min at room temperature. Tubes were centrifuged for 5 min at 17,900 g at 4°C. RNA contained in the aqueous phase was precipitated by addition of 500 µl of isopropanol and incubated for 15 min at room temperature. Tubes were centrifuged for 15 min at 17,900 g at 4°C. RNA pellets were washed with 75% ethanol. Dried RNA pellets were resuspended in DEPC-treated water. RNA concentrations were quantified, and RNA qualities were determined by using Agilent RNA Nano LabChips (Agilent Technologies). RNAs were stored at −80°C until needed.

### cDNA synthesis, fragmentation, labelling and tiling array hybridization

Before cDNA synthesis, RNA integrity from each sample was confirmed on Agilent RNA Nano LabChips (Agilent Technologies). 10 µg of total RNA were reverse transcribed using SuperScript II reverse transcriptase (Invitrogen Life Technologies) and processed following the protocol of the Affymetrix GeneChip Expression Analysis Technical Manual (P/N 702232 Rev. 2) in the presence of 6 ng/ml Actinomycin D to avoid spurious second-strand cDNA synthesis during reverse transcription reaction [64]. Sense RNA corresponding to *B. subtilis* poly-A *lys*, *phe*, *thr*, *trp*, *dap* genes were spiked into sample RNA as control for labelling and hybridization steps. cDNA was digested by DNase I (PIERCE) in 10X DNAse I buffer (USB-Affymetrix) and the size of digestion products was analyzed in the Agilent Bioanalyser 2100 using RNA Nano LabChips to ensure that the fragmentation resulted in a majority of products in the range of 50 to 200 base-pairs. The fragmented cDNA was then biotinylated using terminal deoxynucleotidyl transferase (Promega) and the GeneChip DNA labelling reagent (Affymetrix) following the manufacturer's recommendations. Biotinylated cDNA (5 microgram per array) was hybridized on custom *S. aureus* tiling microarrays designed as described [65]. Hybridization was carried out during 16 h according to the Affymetrix protocol in a total volume of 200 µl per hybridization chamber. Following incubation, the arrays were washed and stained in the Fluidics station 450 (Affymetrix) using the protocol n°FS450_0005. Scanning of the arrays was then performed using the GeneChip scanner 3000 (Affymetrix). A first scan of the chip was carried out with gene expression sub-array parameters followed by a second scan with tiling sub-array parameters. Intensity signals of each probe cells were computed by the GeneChip operating software (GCOS) and stored in cell intensity files (.CEL extension) before preprocessing and analysis.

### Microarray data analysis

Data analysis of the tiling sub-array was performed using the Tiling Analysis Software (TAS) from Affymetrix (http://www.affymetrix.com). Output bar files containing probe signal values were converted in graphic type files (.gr extension file) to be loaded at the *Staphylococcus aureus* transcriptome browser (http://staph.unavarra.es/).

### Construction of cDNA libraries for dRNA-seq, read mapping and statistics analysis

Deep sequencing of RNAs from *S. aureus* 15981 strain was performed as previously described [22]. Mapped reads were included in.wig files to be loaded at the *Staphylococcus aureus* transcriptome browser (http://staph.unavarra.es/).

### Simultaneous mapping of 5′- and 3′-ends of RNA molecules (mRACE)

Simultaneous mapping of 5′- and 3′-ends of the entire *icaR* mRNA molecule and the processing sites was performed by RACE (Rapid Amplification of cDNA Ends) using circularized RNAs as previously described [66], with the following modifications. Specifically, reactions were performed on RNAs extracted from bacteria grown in TSB-gluc until an OD_600 nm_ = 0.8 was reached. Six µg of RNA were treated with TURBO DNase I (Ambion). After phenol extraction to inactivate DNase I, the RNA was divided into two aliquots. Both aliquots were incubated for 45 min at 37°C with the corresponding buffer, in the presence or absence of Tobacco Acid Pyrophosphatase, (TAP) (Epicentre biotechnologies) respectively. This step allows discriminating a 5′-end generated by transcription initiation from a 5′-end provided by RNA processing. The TAP treatment step was avoided when processing sites wanted to be determined. After incubation, acid-phenol and chloroform extractions and ethanol precipitation was performed. Serial dilutions (from 500 ng to 0.5 ng) of the TAP+ and TAP− treated RNAs were prepared. Each dilution was ligated with 40 U of T4 RNA ligase I (New England Biolabs) in the presence of 1X RNA ligase Buffer, 8% DMSO, 10 U of RNase Inhibitor, 1 U of DNase I and RNase-free water in a total volume of 25 µl at 17°C overnight. After acid-phenol and chloroform extractions and ethanol precipitation, the ligated RNAs were resuspended in 10 µl of RNase-free water. RT-PCR reactions were performed using specific outward primers (Table S3) and the SuperScript One-Step RTPCR kit (Invitrogen). RT-PCR products were run on 3% TAE-agarose gels. For mapping of the entire size of the molecule, bands only present in the TAP+ reactions were purified by Gel Extraction kit (QIAGEN) and cloned using TOPO TA Cloning kit (Invitrogen). For mapping of processing sites, all bands observed in the gel were purified and cloned. Eight transformants per cloned band were analysed by PCR using M13 forward and reverse primers. Plasmids containing the expected insert size were sent to sequencing. To determine the localization of the 5′- and 3′-ends, plasmid sequences were compared with *icaR* region from the *S. aureus* 132 genome sequence [67].

### Riboprobes synthesis

Strand-specific riboprobes to detect *icaR* mRNA were synthesized from a PCR product containing a T7 phage promoter sequence (see Table S3 for oligonucleotides). One microgram of these PCR products was used as a matrix template for *in vitro* transcription reaction with phage T7 RNA polymerase, 0.5 mM each ATP, GTP, CTP, and 50 mCi of [α-32P] UTP using the Maxiscript kit (Ambion). The riboprobes were then treated with TURBO DNase I at 37°C for 30 min, and reactions were stopped by addition of 1 µl of 0.5M EDTA. The riboprobes were purified on Bio-Spin 30 columns following the manufacturer's recommendations (Bio-Rad) and were immediately used.

### Northern blots

Northern blots were performed as described [20]. Briefly, 8–15 µg of total RNA were separated in precast 1.25% agarose gels (Sigma) by using 1X NorthernMax MOPS as running buffer (Ambion). After electrophoresis, gels were stained with ethidium bromide and photographed to verify equal loading of RNA samples. Then, RNAs were transferred onto Nytran membranes (0.2 µm pore size) (Sigma) by using NorthernMax One Hour Transfer buffer reagent as described in the manufacturer's protocol (Ambion). RNA was UV cross-linked to the membrane by using the UV Stratalinker 1800 (Stratagene). Membranes were prehybridized for at least 30 min in ULTRAhyb solution (Ambion) at 65°C, followed by addition of labelled strand-specific riboprobe and overnight hybridization at 65°C. Membranes were then washed twice with 2X SSC-0.1% SDS for 5 min at 65°C. The size of the transcripts was estimated by comparison with RNA Millenium molecular weight standards (Ambion). Autoradiography images were registered at different exposition times according to each experiment.

### Chromosomal allelic exchange

To generate the *icaR* 3′-UTR and *yqfR*, (SAOUHSC_01659) deletions, we amplified by PCR two fragments of approximately 500 bp that flanked the left (primers A and B, Table S3) and right sequences (primers C and D, Table S3) of the region targeted for deletion. The PCR products were amplified with Phusion High-Fidelity DNA Polymerase (Fermentas-Thermo Scientific), purified and cloned separately in pCR-Blunt II TOPO vector (Invitrogen). Fragments were then fused by ligation into the shuttle vector pMAD [68]. The resulting plasmid was transformed into *S. aureus* 15981 or 132 strains by electroporation. To generate *hfq* deletion pLUG533 plasmid was used [36]. Homologous recombination experiments were performed as described [69]. Erythromycin sensitive white colonies, which no longer contained the pMAD plasmid, were tested by PCR using primers E and F and DNA sequencing.

### Plasmid constructions

All PCR fragments were amplified from *S. aureus* 132 chromosomal DNA using Phusion High-Fidelity DNA Polymerase (Fermentas-Thermo Scientific) and the appropriate oligonucleotides. PCR fragments were purified and cloned into pCR-Blunt II TOPO vector (Invitrogen). DNA fragments were excised from this vector with appropriate restriction enzymes and then subcloned into the shuttle vectors pSA14 [70] or pCN40 [71]. pSA14 plasmid is a pMK4 derivative carrying promoterless *E. coli lacZ* gene for constructing transcriptional fusions while pCN40 plasmid allows the expression of the gene of interest from the constitutive P*_blaZ_* promoter. To construct pSA14-P*ica* plasmid, a 422 bp DNA fragment, which includes the *ica* operon promoter, was amplified with icaA-pSA14-Fw and icaA-pSA14-Rv oligonucleotides. pCN40 plasmid derivatives which expressed different *icaR* mRNA versions from the constitutive P*blaZ* promoter were constructed as follows. pIcaRm plasmid was constructed by amplifying a PCR fragment of 1,079 nt with IcaR+1 and IcaR-Term oligonucleotides and cloning into the BamHI/EcoRI site of pCN40. pIcaRmΔ3′UTR plasmid was constructed by amplifying a PCR fragment of 748 nt with IcaR+1 and IcaR-Term oligonucleotides using *S. aureus* 132 Δ3′-UTR chromosomal DNA as template and cloning into BamHI/EcoRI sites of pCN40. To construct p^FLAG^IcaRm_WT, p^FLAG^IcaRmΔ3′UTR, pIcaRmΔanti-SD, pIcaRm_SUBST, p^FLAG^IcaRmΔanti-SD, p^FLAG^IcaRm_SUBST and pIcaRm-Compensatory plasmids, overlapping PCR performed with oligonucleotides shown in Table S3 was used. All constructed plasmids were confirmed by sequencing.

### Quantitative reverse transcription PCR

Total RNA from bacterial cells grown until OD_600 nm_ of 0.8 was extracted as described above. Each RNA sample was subjected to TURBO DNase I (Ambion) treatment for 30 min at 37°C. The enzyme was inactivated by phenol-chloroform extractions. RNA quality was assessed with an Agilent 2100 Bioanalyzer. Twenty µl of random primers (50 ng µl^−1^; Invitrogen) and 20 µl of deoxynucleoside triphosphates (dNTPs) (10 mM mix; Invitrogen) were added to the samples containing 8 to 10 µg of RNA in a volume of 100 µl of diethyl pyrocarbonate (DEPC) water. After 5 min of incubation at 65°C, samples were chilled on ice at least during 1 min, and a reverse transcription (RT) mix containing 44 µl of 5X first-strand buffer (Invitrogen), 22 µl of dithiothreitol (DTT) (0.1 M; Invitrogen), 2 µl of SuperScript III Retrotranscriptase (200 U µl^−1^; Invitrogen) and 1 µl of RNase Out (40 U µl^−1^; Invitrogen) was added to each preparation. cDNA was obtained after a cycle of 10 min at 25°C, 50 min at 50°C, and 5 min at 85°C. RNA was eliminated by the addition of 1 µl of RNase H (10 U µl^−1^; Invitrogen) and incubation for 20 min at 37°C. cDNA samples were purified with CentriSep spin columns (Princeton separations). cDNA concentration was adjusted to 100 ng µl^−1^. One µl of the cDNA samples was used for real-time quantitative PCR using SYBR green PCR master mix (Applied Biosystems) and the ABI Prism 7900 HT instrument (Applied Biosystems). The PCR was performed under the following conditions: 95°C for 20 s, 40 cycles of 95°C for 1 s and 60°C for 20 s, and a final step at 95°C for 15 s, 60°C for 15 s, and 95°C for 15 s. *icaR* and *gyrB* mRNA levels were quantified by cDNA amplification using oligonucleotides described in Table S3 and values were normalized to those of the housekeeping *gyrB* gene.

### Western blot analysis

Overnight cultures of the strains tested were diluted 1∶100 in TSB-gluc, and 20 ml of this cell suspension were grown in 125 ml flasks until OD_600 nm_ reached 0.8. Ten ml of bacterial cultures were centrifuged and pellets were resuspended in 100 µl PBS. Then, 2 µl of Lysostaphin 1 mg ml^−1^ (Sigma) and 3 µl of DNase I 1 mg ml^−1^ (Sigma) were added. After 2 h of incubation at 37°C cell lysates were centrifuged and supernatants were collected. Protein concentration was determined with the Bio-Rad protein assay (Bio-Rad). Samples were adjusted to 5–10 µg µl^−1^ of total protein and one volume of Laemmli buffer was added. Total protein extracts were denatured by boiling at 100°C for 5 min. Proteins were separated on 12% SDS-polyacrylamide gels and stained with 0.25% Coomassie brilliant blue R250 (Sigma) as loading controls. For Western blotting, proteins were transferred onto Hybond-ECL nitrocellulose membranes (Amersham Biosciences) by semi-dry electroblotting. Membranes were blocked overnight with 5% skimmed milk in phosphate-buffered saline (PBS) with 0.1% Tween 20, and incubated with anti-FLAG antibodies labelled with phosphatase alkaline (Sigma) diluted 1∶500 for 2 h at room temperature. 3XFLAG labelled IcaR protein was detected with the SuperSignal West Pico Chemiluminescent Substrate (Thermo Scientific) following the manufacturer's recommendations.

### mRNA stability assays

Overnight cultures were diluted 1∶100 in TSB-gluc, and 150 ml of these cell suspensions were grown in 500 ml flasks until OD_600 nm_ = 0.8 was reached. Twelve ml of cultures were transferred to six sterile 15 ml falcon tubes containing 300 µg ml^−1^ Rifampicin. The tube corresponding to time 0 min also contained 2.5 mL STOP solution (5% phenol equilibrated at pH 7 and 95% of ethanol). After addition of the culture, the time “0 min” tube was immediately centrifuged at 4,500 rpm during 3 min, the supernatant discarded and the pellet frozen in liquid nitrogen. The rest of the tubes were incubated at 37°C and 2.5 ml of STOP solution were added at times 2, 4, 8, 15 and 30 min after rifampicin addition respectively. Then, each tube was centrifuged at 4,500 rpm during 3 min. Supernatants were discarded and pellets were frozen in liquid nitrogen and stored at −80°C until needed. RNA extractions and Northern blots were performed as described above.

### Visualization of *icaR* mRNA dimers on agarose gel electrophoresis

The full-length *icaR* mRNA was produced by *in vitro* transcription using T7 RNA polymerase. To visualize the monomeric form of the mRNA, *icaR* mRNA (0.25 µg) was denatured 3 min at 90°C in 10 µl of sterile bi-distillated water or of a buffer containing Tris-HCl 20 mM pH 7.5, 1 mM EDTA, chilled on ice for 1 min followed by an incubation at 37°C for 15 min. To visualize alternative conformations of *icaR* mRNA, the mRNA (0.25 µg) was first denatured in 8 µl of sterile bi-distillated water for 3 min at 90°C, chilled on ice, and renatured at 37°C for 15 min by adding 2 µl of a 5X concentrated buffer containing Tris-HCl 100 mM pH 7.5, 250 mM KCl in the absence or in the presence of 50 mM MgCl_2_. All samples were then mixed with 2 µl of loading buffer (48% glycerol, 0.01% bromophenol blue) and electrophoresed on 1% agarose gel in 0.5X TBE buffer. The RNA was then visualized after ethidium bromide staining.

### Gel shift assays

5′-UTR and 3′-UTR wild type PCR fragments (117 and 120 nt respectively) were amplified using chromosomal DNA from *S. aur*eus 132 strain, while 5′-UTR-compensatory and 3′-UTR-subsituted fragments were amplified from the corresponding plasmids using oligonucleotides shown in Table S3. These PCR fragments were used as templates for T7 *in vitro* transcription of RNA fragments using Riboprobe *in vitro* Transcription System (Promega). When needed 50 mCi of [α-32P] UTP was used for radiolabelling. Then RNA fragments were purified by electrophoresis on an 8.3 M urea/6% polyacrylamide gel. The bands were excised from the gel and RNA fragments were eluted with elution buffer (3 M ammonium acetate pH 5.2, 1mM EDTA, 2.5% (v/v) phenol pH 4.3) overnight at room temperature. RNA fragments were ethanol precipitated, resuspended in RNase free water and quantified by Biophotometer Plus (Eppendorf). The yield of the labelled substrates (cpm µl^−1^) was determined by scintillation counting.

Binding assays were performed in 1X TMN buffer (20 mM Tris acetate pH7.6, 100 mM sodium acetate, 5 mM magnesium acetate) as previously described [72]. Briefly, labelled RNA fragments (0.025 pmol of 5′-UTR-WT or 5′-UTR-compensatory) were incubated with increasing concentrations of unlabelled RNA fragments (3′-UTR WT or 3′-UTR-substituted) in a total volume of 10 µl, at 37°C for 30 min. Binding reactions were then mixed with 2 µl of loading buffer (48% glycerol, 0.01% bromophenol blue) and electrophoresed on native 5% polyacrylamide gels in 0.5X TBE buffer at 200 V in a cold room for 3 h. Gels were analysed using a PhosphorImager (Molecular Dynamics).

### Purification of recombinant *S. aureus* RNase III

*S. aureus rnc* gene was amplified with primer RNAse III Fw (NdeI) and RNAse III Rv (BamHI) (Table S3). The purified PCR fragment was double digested with BamHI and NdeI and ligated into the pET-15b vector (Promega), generating plasmid pET-15b RNAse III. This plasmid was transformed in *E. coli* BL21(DE3) *rnc*105 *recA*. This strain is slow growing but allows overproduction of His_6_-RNase III [73]. The BL21(DE3) *rnc*105 *recA* strain carrying the pET-15b RNAse III plasmid was grown in 200 ml of LB medium supplemented with ampicillin (100 µg ml^−1^) to an OD_600 nm_ of 1.5. At this point, protein expression was induced by addition of 1 mM IPTG and the culture was further incubated overnight at 15°C. Cells were then harvested by centrifugation, the pellet was washed with 12 ml of cold buffer (25 mM Tris-HCl pH 8.0, 8% ammonium sulphate, 0.1 mM EDTA) and resuspended in 6 ml of the same buffer. Cells were lysed using a French Press at 900 psi in the presence of 0.1 mM of PMSF. After lysis, the crude extracts were treated with 125 U of Benzonase (Sigma). The protein extract was then clarified by centrifugation for 30 min, 27,000 g at 4°C. The recombinant histidine tagged RNase III was purified by affinity chromatography, using the ÄKTA FPLCTM System (GE Healthcare). The clarified extracts were loaded into a HisTrap HP Sepharose 1 ml column. Protein elution was achieved with a linear imidazole gradient (from 0 mM to 300 mM) in buffer B (25 mM Tris-HCl pH 8.0, 1M NH_4_Cl, 300 mM imidazole). Fractions containing the protein of interest, free of contaminants, were pooled and buffer exchanged by dialysis against Desalting Buffer (25 mM Tris-HCl pH 8.0, 500 mM KCl, 0,1 mM DTT, 50% glycerol) using a Slide-A-Lyzer Dialysis Cassette (Thermo Scientific) with a molecular mass cut-off of 10 kDa. Protein samples were quantified using the Bio-Rad protein assay (Bio-Rad) and stored at −20°C. The purity of the enzyme was analysed by SDS-PAGE.

### RNase III activity assays

RNA substrates were prepared as described in the Gel shift assays section. The activity of purified RNase III was tested over [^32^P]-α-UTP labelled 5′-UTR-WT fragment in the presence or absence of 3′-UTR-WT or 3′-UTR-substituted fragments. Hybridizations between labelled and unlabelled substrates were performed in a 1∶50 molar ratio in the Tris component of the activity buffer by incubation for 10 minutes at 80°C, followed by 45 minutes at 37°C. The same treatment was applied to free 5′-UTR-WT. Activity assays were carried out in a final volume of 40 µl containing the activity buffer (30 mM Tris-HCl pH 8, 160 mM NaCl and 0.1 mM DTT) and approximately 0.14 pmol of substrate. 10 mM MgCl_2_ were added to the reaction mixture. As a control, an aliquot (without the enzyme) was taken and incubated in the same conditions until the end of the assay. Reactions were started by the addition of the enzyme at a concentration of 500 nM and were incubated at 37°C [74]. Samples were withdrawn at different times and reactions were stopped by the addition of formamide-containing dye supplemented with 10 mM EDTA. Reaction products were run in a 7M urea/10% polyacrylamide gel, visualized by PhosphorImager and analysed using ImageQuant software (Molecular Dynamics).

### Toeprinting assays

Full length *icaR* mRNA or its variants were cloned [75] into StuI and BamHI restriction sites of pUT7 plasmid for *in vitro* transcription. The whole 5′-UTR including 72-nt of the ORF and the 3′-UTR (186 nt) were transcribed from PCR products obtained with oligos T7 5′-UTR and IcaR 5′ rev, and T7 3′-UTR and IcaR 3′ rev, respectively (Table S3). *S. aureus* 30S ribosomal subunits were prepared as described [76]. The formation of a simplified translation initiation complex with mRNAs and the extension conditions were as described [76]. Standard conditions contained 5 nM *icaR* mRNA or *icaR* 5′-UTR annealed to a 5′ end labeled oligonucleotide (IcaR 5′ rev), 500 nM *S. aureus* 30S ribosomal subunits, and 0.5 to 10 µM of *icaR* 3′-UTR in 10 µl of buffer containing 20 mM Tris-acetate pH 7.5, 60 mM NH_4_Cl, 8.5 mM magnesium acetate and 1 mM DTT. The 30S subunits were renatured for 10 min at 37°C before incubation with the mRNAs. After 10 min of incubation at 37°C, the initiator tRNA (1 µM) was added, and the reaction was further incubated for 5 min at 37°C. Reverse transcription was conducted with one unit of AMV reverse transcriptase for 15 min at 15°C. Toeprint was run on 8% polyacrylamide gels and visualized by autoradiography.

### PIA-PNAG quantification

Cell surface PIA/PNAG exopolysaccharide levels were quantified as previously described [29]. Briefly, overnight cultures of the strains tested were diluted 1∶40 in the appropriate medium and 2 ml of this cell suspension were used to inoculate sterile 24-well polystyrene microtiter plates (Sarstedt). After 24 h of static incubation at 37°C, the same number of cells of each strain was resuspended in 50 µl of 0.5 M EDTA (pH 8.0). Then, cells were incubated for 5 min at 100°C and centrifuged 17,000 g for 5 min. Each supernatant (40 µl) was incubated with 10 µl of proteinase K (20 mg ml^−1^) (Sigma) for 30 min at 37°C. After the addition of 10 µl of Tris-buffered saline (20 mM Tris-HCl, 150 mM NaCl [pH 7.4]) containing 0.01% bromophenol blue, 5 µl were spotted on a nitrocellulose membrane using a Bio-Dot microfiltration apparatus (Bio-Rad). The membrane was blocked overnight with 5% skimmed milk in phosphate-buffered saline (PBS) with 0.1% Tween 20, and incubated for 2 h with specific anti-PNAG antibodies diluted 1∶10,000 [77]. Bound antibodies were detected with peroxidase-conjugated goat anti-rabbit immunoglobulin G antibodies (Jackson ImmunoResearch Laboratories, Inc., Westgrove, PA) diluted 1∶10,000 and developed using the SuperSignal West Pico Chemiluminescent Substrate (Thermo Scientific).

### Biofilm formation assay

To analyze biofilm formation under flow conditions, we used 60-ml microfermentors [78] (Pasteur Institute; (www.pasteur.fr/recherche/unites/Ggb/biofilmfermenter.html) with a continuous flow of 40 ml of TSB h^−1^ and constant aeration with sterile pressed air as previously described [69]. Submerged Pyrex slides served as the growth substratum. 10^8^ bacteria from an overnight preculture grown in TSB-gluc of each strain were used to inoculate microfermenters and were cultivated 8 h at 37°C. Biofilm development was recorded with a digital camera.

## Supporting Information

Figure S1 Examples of *S. aureus* transcripts carrying long 3′-UTRs. Drawings are IGB software images showing tiling array signals and RNA-seq mapped reads. The exterior tracks correspond to the tiling signals of four unrelated staphylococcal strains, 15981 (black line), ISP479r (green line), RN10359 (red line) and MW2 (blue line) while the interior tracks (black) correspond to the mapped reads from *S. aureus* 15981 strain. Blue boxes, ORFs; red boxes, intrinsic transcriptional terminators; Green dash lines, transcript. One representative example of a (A) *bona fide* long 3′-UTR; (B) a terminating-read-through dependent long 3′-UTR; (C) a riboswitch-dependent long 3′-UTR.
(PDF)

Figure S2 IGB software image showing tiling arrays signals and RNA-seq mapped reads distribution in the *icaRADBC locus*. The exterior tracks correspond to tiling signals of four unrelated staphylococcal strains, 15981 (black line), ISP479r (green line), RN10359 (red line) and MW2 (blue line) while the interior tracks (black) correspond to the mapped reads from *S. aureus* 15981 strain. Blue boxes, ORFs; red boxes, intrinsic transcriptional terminators.
(PDF)

Figure S3 *icaR* as well as *hld* 3′-UTRs are highly conserved in *S. aureus*. Nucleotide variation rate was calculated using the 176 *S. aureus* genomic sequences available at NCBI web page. Vertical lines represent a nucleotide change in at least one *S. aureus* genome. (A) *icaR* mRNA region. (B) *hld* mRNA (RNAIII) region. ORF, open reading frame; IGR, intergenic region comprised between the corresponding 3′-end and the end of the next known transcript.
(PDF)

Figure S4 Schematic representation of the RNA secondary structure prediction of the whole *icaR* mRNA molecule generated by Mfold program (http://mfold.rit.albany.edu/) [35]. G-U paring was allowed. The 5′-3′-UTRs interaction region is amplified to show pairing nucleotides. The lowest-energy secondary-structural prediction is shown. RNA stem-loops were organized to avoid image superposition.
(PDF)

Figure S5 Interaction of two *icaR* mRNA molecules in *trans*. Native agarose gel electrophoresis of the full-length *icaR* mRNA synthetized *in vitro*. Line 1, RNA in water; line 2, RNA in TE buffer (Tris-HCl 20 mM p 7.5, 1 mM EDTA) incubated 3 min at 90°C, chilled on ice and incubated 15 min at 37°C; line 3, RNA renatured in a buffer containing 20 mM Tris HCl pH 7.5, 50 mM KCl at 37°C for 15 min; line 4, RNA in a buffer containing 20 mM Tris HCl pH 7.5, 50 mM KCl, 10 mM MgCl_2_ renatured at 37°C for 15 min. Sizes of some bands of the molecular weight marker (M) are indicated.
(PDF)

Figure S6 Toeprinting assays performed with *S. aureus spa* mRNA. (A) Sequence of the 5′-UTR of *spa* and *icaR* mRNA are given for comparison. The Shine-Dalgarno (SD) sequence is in bold letter as well as the initiation codon UUG. (B) Autoradiography showing the toeprinting assay performed on *spa* mRNA. The toeprint at position +16, representing the formation of ternary initiation complex formed by *S. aureus* 30S ribosomal subunit, mRNA and the initiator tRNA_f_^Met^, is indicated. (C) Comparison of the toeprint signals from panel B and Figure 7C. Band intensity was first normalized according to the full-length extension product bands using the SAFA software [79], and the toeprint signal is given in %.
(PDF)

Figure S7 IcaR protein levels expressed from wild type and UCCCC substituted mRNAs in wild type and *rnc* mutant strains. A representative Western blot showing IcaR protein levels in wild type and *rnc* deleted strains constitutively expressing the *icaR* mRNA wild type or the mRNA carrying the UCCCCUG substitution. The 3XFLAG tagged IcaR protein was detected with commercial anti-3XFLAG antibodies. A Coomassie stained gel portion is shown as loading control.
(PDF)

Table S1 Strains used in this study.
(PDF)

Table S2 Plasmids used in this study.
(PDF)

Table S3 Oligonucleotides used in this study.
(PDF)
