# Supplementary figures and images for: Base Pairing Interaction between 5′- and 3′-UTRs Controls icaR mRNA Translation in Staphylococcus aureus
Source: PLoS Genet. 2013 Dec 19;9(12):e1004001. doi: 10.1371/journal.pgen.1004001 (PMC3868564; doi:10.1371/journal.pgen.1004001)

### A ■ *Bona fide* long 3'-UTR

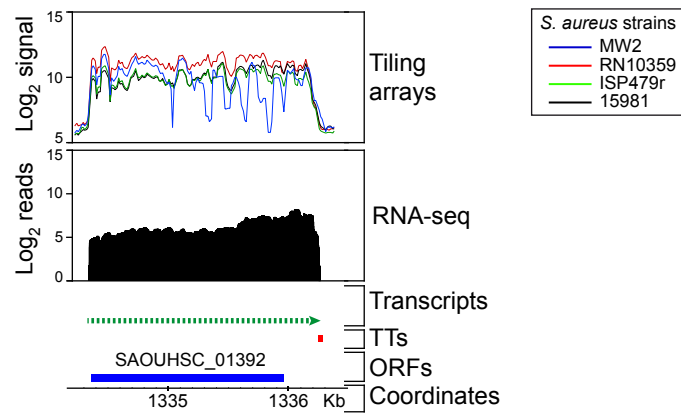

### B ■ Terminating-read-through dependent long 3'-UTR

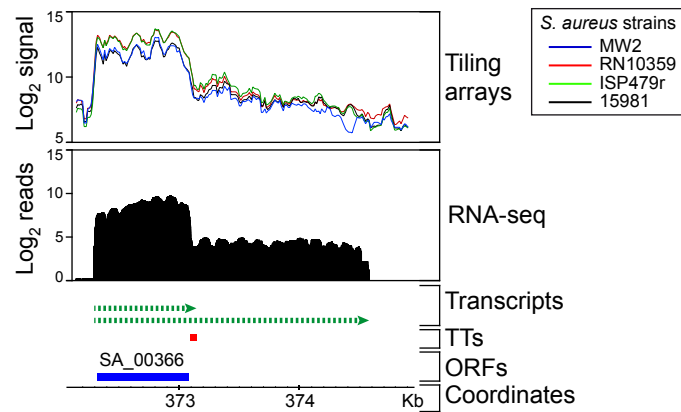

### C ■ Riboswitch-dependent 3'-UTR

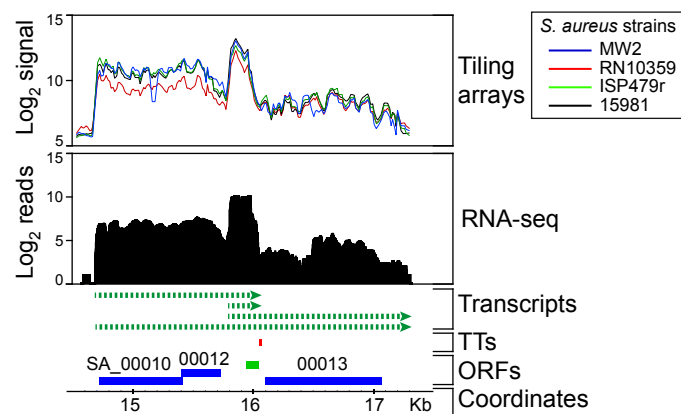

Supplement: Figure S1 — Examples of S. aureus transcripts carrying long 3′-UTRs. Drawings are IGB software images showing tiling array signals and RNA-seq mapped reads. The exterior tracks correspond to the tiling signals of four unrelated staphylococcal strains, 15981 (black line), ISP479r (green line), RN10359 (red line) and MW2 (blue line) while the interior tracks (black) correspond to the mapped reads from S. aureus 15981 strain. Blue boxes, ORFs; red boxes, intrinsic transcriptional terminators; Green dash lines, transcript. One representative example of a (A) bona fide long 3′-UTR; (B) a terminating-read-through dependent long 3′-UTR; (C) a riboswitch-dependent long 3′-UTR. (PDF) [file pgen.1004001.s001.pdf]

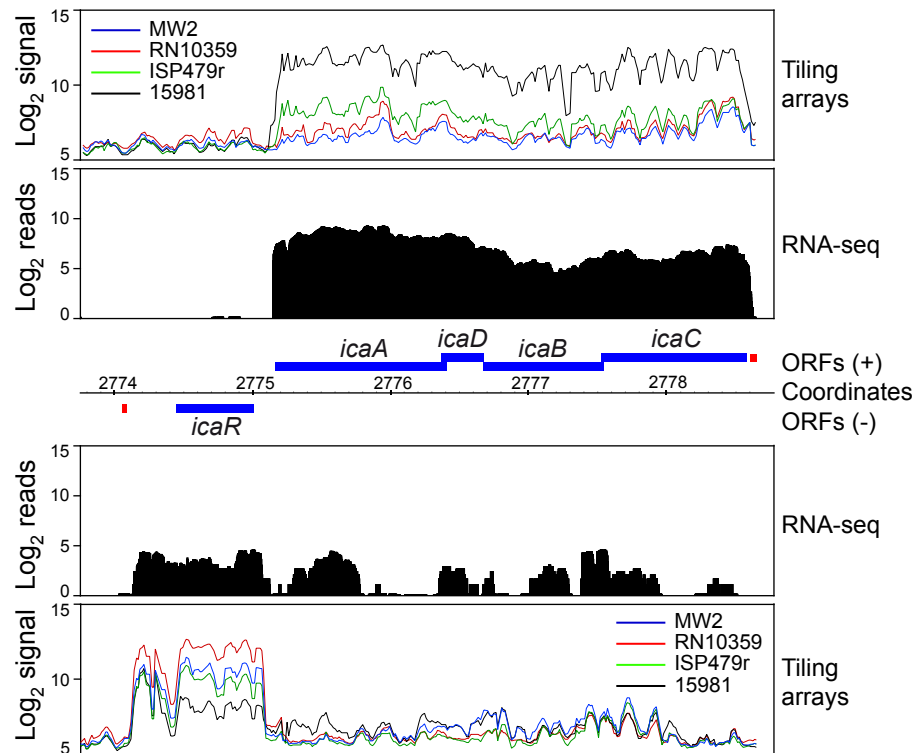

Supplement: Figure S2 — IGB software image showing tiling arrays signals and RNA-seq mapped reads distribution in the icaRADBC locus. The exterior tracks correspond to tiling signals of four unrelated staphylococcal strains, 15981 (black line), ISP479r (green line), RN10359 (red line) and MW2 (blue line) while the interior tracks (black) correspond to the mapped reads from S. aureus 15981 strain. Blue boxes, ORFs; red boxes, intrinsic transcriptional terminators. (PDF) [file pgen.1004001.s002.pdf]

**A** *icaR* mRNA

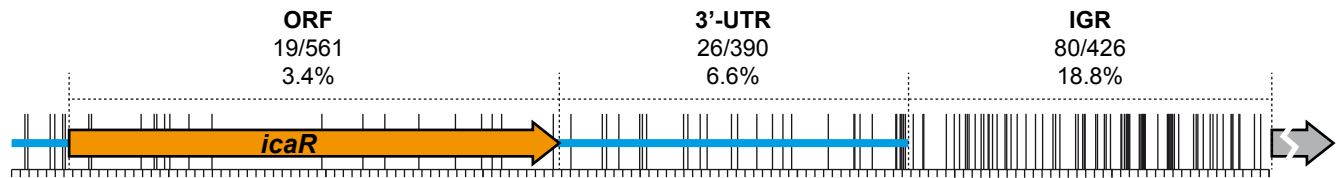

**B** *hld* mRNA (RNAIII)

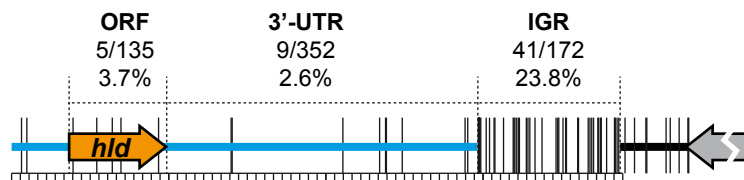

Supplement: Figure S3 — icaR as well as hld 3′-UTRs are highly conserved in S. aureus. Nucleotide variation rate was calculated using the 176 S. aureus genomic sequences available at NCBI web page. Vertical lines represent a nucleotide change in at least one S. aureus genome. (A) icaR mRNA region. (B) hld mRNA (RNAIII) region. ORF, open reading frame; IGR, intergenic region comprised between the corresponding 3′-end and the end of the next known transcript. (PDF) [file pgen.1004001.s003.pdf]

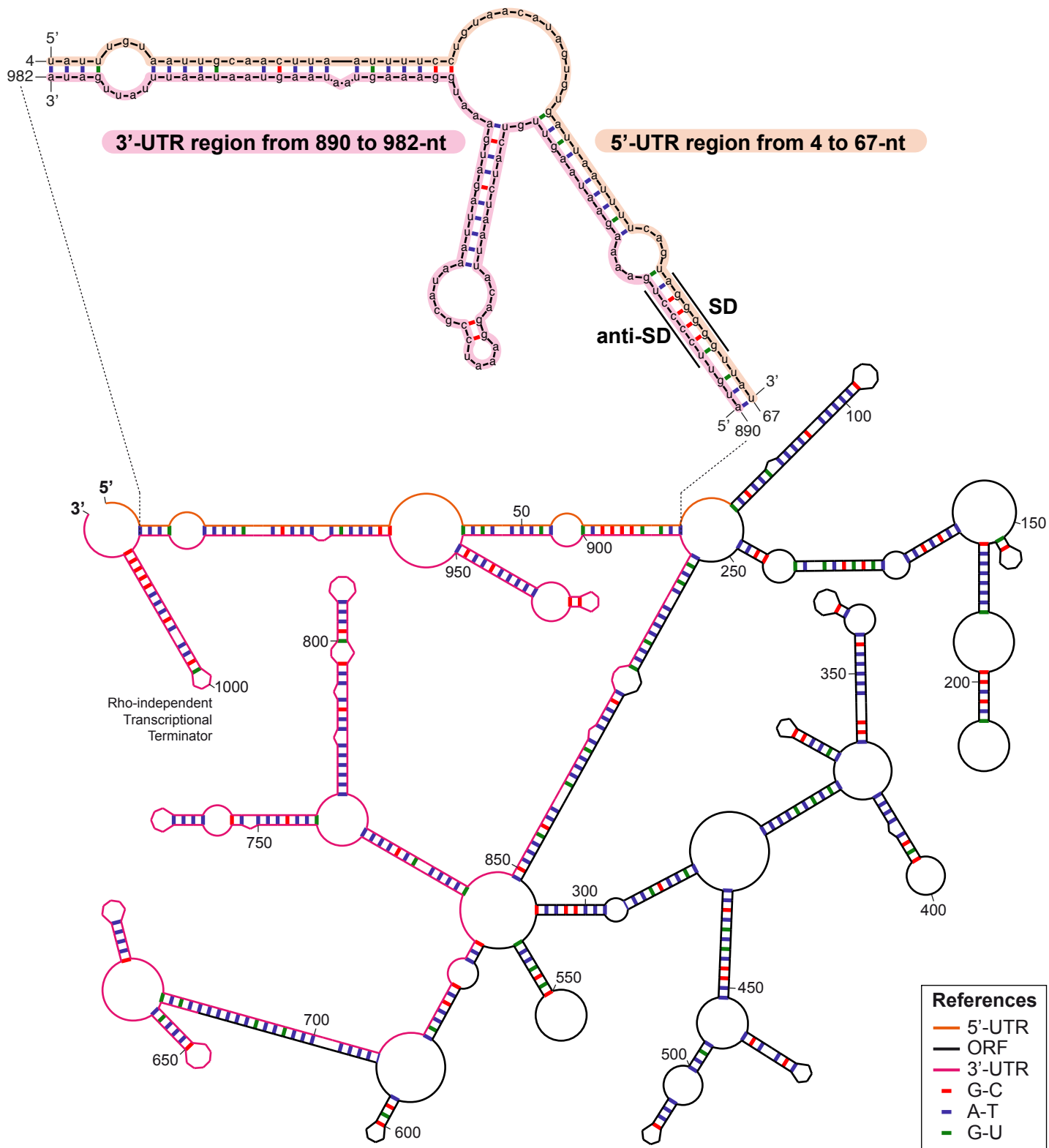

Supplement: Figure S4 — Schematic representation of the RNA secondary structure prediction of the whole icaR mRNA molecule generated by Mfold program (http://mfold.rit.albany.edu/) [35]. G-U paring was allowed. The 5′-3′-UTRs interaction region is amplified to show pairing nucleotides. The lowest-energy secondary-structural prediction is shown. RNA stem-loops were organized to avoid image superposition. (PDF) [file pgen.1004001.s004.pdf]

Full length *icaR* mRNA

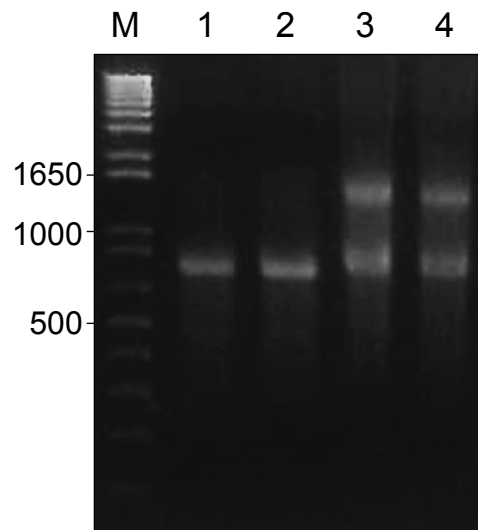

Supplement: Figure S5 — Interaction of two icaR mRNA molecules in trans. Native agarose gel electrophoresis of the full-length icaR mRNA synthetized in vitro. Line 1, RNA in water; line 2, RNA in TE buffer (Tris-HCl 20 mM p 7.5, 1 mM EDTA) incubated 3 min at 90°C, chilled on ice and incubated 15 min at 37°C; line 3, RNA renatured in a buffer containing 20 mM Tris HCl pH 7.5, 50 mM KCl at 37°C for 15 min; line 4, RNA in a buffer containing 20 mM Tris HCl pH 7.5, 50 mM KCl, 10 mM MgCl2 renatured at 37°C for 15 min. Sizes of some bands of the molecular weight marker (M) are indicated. (PDF) [file pgen.1004001.s005.pdf]

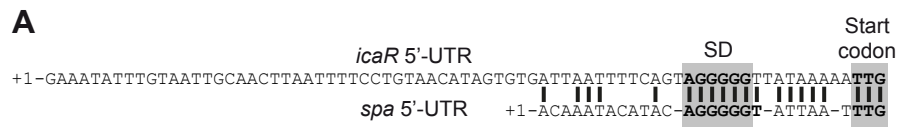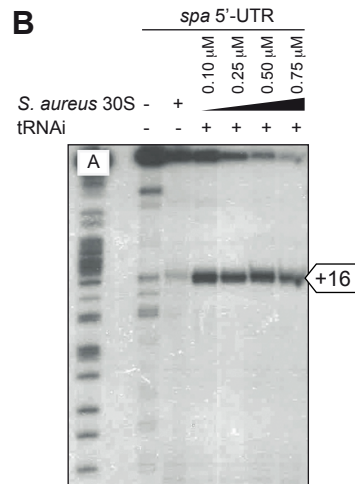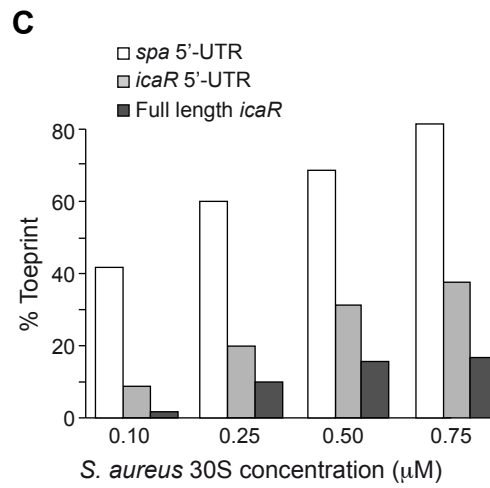

Supplement: Figure S6 — Toeprinting assays performed with S. aureus spa mRNA. (A) Sequence of the 5′-UTR of spa and icaR mRNA are given for comparison. The Shine-Dalgarno (SD) sequence is in bold letter as well as the initiation codon UUG. (B) Autoradiography showing the toeprinting assay performed on spa mRNA. The toeprint at position +16, representing the formation of ternary initiation complex formed by S. aureus 30S ribosomal subunit, mRNA and the initiator tRNAf Met, is indicated. (C) Comparison of the toeprint signals from panel B and Figure 7C. Band intensity was first normalized according to the full-length extension product bands using the SAFA software [79], and the toeprint signal is given in %. (PDF) [file pgen.1004001.s006.pdf]

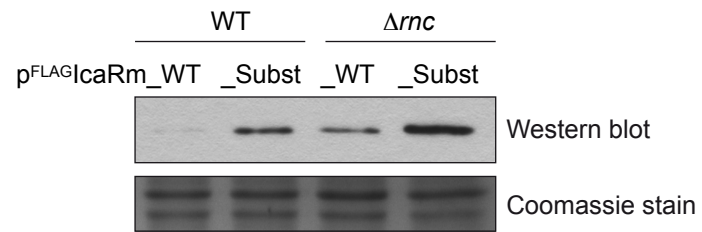

Supplement: Figure S7 — IcaR protein levels expressed from wild type and UCCCC substituted mRNAs in wild type and rnc mutant strains. A representative Western blot showing IcaR protein levels in wild type and rnc deleted strains constitutively expressing the icaR mRNA wild type or the mRNA carrying the UCCCCUG substitution. The 3XFLAG tagged IcaR protein was detected with commercial anti-3XFLAG antibodies. A Coomassie stained gel portion is shown as loading control. (PDF) [file pgen.1004001.s007.pdf]
