# Supplementary material for: Base Pairing Interaction between 5′- and 3′-UTRs Controls icaR mRNA Translation in Staphylococcus aureus
Source: PLoS Genet. 2013 Dec 19;9(12):e1004001. doi: 10.1371/journal.pgen.1004001 (PMC3868564; doi:10.1371/journal.pgen.1004001)
Supplement: Table S1 — Strains used in this study. (PDF) [file pgen.1004001.s008.pdf]

Table S1. Strains used in this study.

Strains	Relevant characteristic(s)	MIC[a]	Source or reference[b]
Staphylococcus aureus			
15981	Clinical strain. Biofilm positive in TSBg	532	[1]
MW2	Typical community-acquired strain of MRSA, which was isolated in 1998 in North Dakota, USA.	3566	NRS123, [2]
RN10359	RN450 lysogenic for 80α phage	3337	[3]
ISP479r	ISP479c with *rsbU* gene restored	1680	[4]
132	MRSA clinical strain with the capacity to form proteinaceous biofilm in TSB-glucose and PIA-PNAG-dependent biofilm in TSB-NaCl	29	[5]
15981 Δ3'-UTR	15981 strain with deletion in the *icaR* 3' UTR	2695	This study
132 Δ3'-UTR	132 strain with deletion in the *icaR* 3' UTR	3661	This study
15981 ^{FLAG}IcaR	15981 strain expressing the 3XFLAG tagged IcaR protein from the chromosome	2653	This study
15981 ^{FLAG}IcaR Δ3'-UTR	15981 Δ3'-UTR strain expressing the 3XFLAG tagged IcaR protein from the chromosome	2649	This study
15981 pCN40	15981 strain carrying the pCN40 plasmid	1014	This study
15981 p^{FLAG}IcaRm	15981 strain carrying the p^{FLAG}IcaRm plasmid	2683	This study
15981 p^{FLAG}IcaRmΔ3'UTR	15981 strain carrying the p^{FLAG}IcaRmΔ3'UTR plasmid	2684	This study
15981 p^{FLAG}IcaRmΔanti-SD	15981 strain carrying the p^{FLAG}IcaRmΔanti-SD plasmid	4392	This study
15981 p^{FLAG}IcaRm_SUBST	15981 strain carrying the p^{FLAG}IcaRm-SUBST-anti-SD plasmid	4395	This study
15981 pIcaRm	15981 strain carrying the pIcaRm plasmid	1011	This study
15981 pIcaRmΔ3'UTR	15981 strain carrying the pIcaRmΔ3'UTR plasmid	1012	This study
15981 pIcaRmΔanti-SD	15981 strain carrying the pIcaRmΔanti-SD plasmid	4391	This study
15981 pIcaRm_SUBST	15981 strain carrying the pIcaRm-SUBST-anti-SD plasmid	4394	This study
15981 Δ*rnc::cat86*	15981 strain with deletion in the *rnc* gene	1327	[6]
15981 Δ*pnp*	15981 strain with deletion in the *pnp* gene	1100	[6]
15981 Δ*yqfR*	15981 strain with deletion in the *SA1387* gene which encodes a DEAD-box RNA helicase	1394	This study
15981 Δ*hfq::cat86*	15981 strain with deletion in the *hfq* gene	1055	This study
15981 Δ*rnc* p^{FLAG}IcaRm	15981 Δ*rnc::cat86* strain carrying the p^{FLAG}IcaRm plasmid	2706	This study
15981 Δ*rnc* p^{FLAG}IcaRmΔ3'UTR	15981 Δ*rnc::cat86* strain carrying the p^{FLAG}IcaRmΔ3'UTR plasmid	2707	This study
15981 Δ*rnc* p^{FLAG}IcaRm_SUBST	15981 Δ*rnc::cat86* strain carrying the p^{FLAG}IcaRm-SUBST-anti-SD plasmid	4970	This study
15981 Δ*yqfR* p^{FLAG}IcaRm	15981 Δ*yqfR* strain carrying the p^{FLAG}IcaRm plasmid	2704	This study
15981 Δ*hfq* p^{FLAG}IcaRm	15981 Δ*hfq::cat86* strain carrying the p^{FLAG}IcaRm plasmid	2708	This study
15981 pSA14-PicaA53	15981 strain carrying the pSA14-PicaA53 plasmid	1449	This study
Escherichia coli strains			
XL1 Blue	Used for cloning experiments		Stratagene
XL1 Blue pCN40	XL1Blue carrying the pCN40 plasmid from RN9594 (NRS594)	2648	This study
BL21(DE3) rnc105	BL21(DE3) *rnc105 recA*	4848	[7]
BL21 pET-15b RNase III	BL21(DE3) *rnc105 recA* strain carrying the pET-15b expressing the *S. aureus* RNase III fused to His-tag	4767	This study

[a]Microbial Biofilm Laboratory strain collection number.

[b]References

1. Valle J, Toledo-Arana A, Berasain C, Ghigo J-M, Amorena B, et al. (2003) SarA and not sigmaB is essential for biofilm development by *Staphylococcus aureus*. Mol Microbiol 48: 1075–1087.
2. Baba T, Takeuchi F, Kuroda M, Yuzawa H, Aoki K-I, et al. (2002) Genome and virulence determinants of high virulence community-acquired MRSA. Lancet 359: 1819–1827.
3. Ubeda C, Barry P, Penadés JR, Novick RP (2007) A pathogenicity island replicon in *Staphylococcus aureus* replicates as an unstable plasmid. Proc Natl Acad Sci USA 104: 14182–14188.
4. Toledo-Arana A, Merino N, Vergara-Irigaray M, Débarbouillé M, Penadés JR, et al. (2005) *Staphylococcus aureus* develops an alternative, *ica*-independent biofilm in the absence of the *arlRS* two-component system. Journal of Bacteriology 187: 5318–5329.
5. Vergara-Irigaray M, Valle J, Merino N, Latasa C, García B, et al. (2009) Relevant role of fibronectin-binding proteins in *Staphylococcus aureus* biofilm-associated foreign-body infections. Infection and Immunity 77: 3978–3991.
6. Lasa I, Toledo-Arana A, Dobin A, Villanueva M, de los Mozos IR, et al. (2011) Genome-wide antisense transcription drives mRNA processing in bacteria. Proc Natl Acad Sci USA 108: 20172–20177.
7. Amarasinghe AK, Calin-Jageman I, Harmouch A, Sun W, Nicholson AW (2001) *Escherichia coli* ribonuclease III: affinity purification of hexahistidine-tagged enzyme and assays for substrate binding and cleavage. Meth Enzymol 342: 143–158.
