# Supplementary material for: Base Pairing Interaction between 5′- and 3′-UTRs Controls icaR mRNA Translation in Staphylococcus aureus
Source: PLoS Genet. 2013 Dec 19;9(12):e1004001. doi: 10.1371/journal.pgen.1004001 (PMC3868564; doi:10.1371/journal.pgen.1004001)
Supplement: Table S2 — Plasmids used in this study. (PDF) [file pgen.1004001.s009.pdf]

Table S2. Plasmids used in this study.

Plasmids	Relevant characteristic(s)	Source or reference[a]
pMAD	*E. coli* - *S. aureus* shuttle vector with a thermosensitive origin of replication for Gram-positive bacteria. The vector contains the *bgaB* gene encoding a β- galactosidase under the control of a constitutive promoter as reporter of plasmid presence. Ap^R, Em^R.	[1]
pMAD Δ3'UTR	pMAD plasmid containing the mutant allele for deletion of the long 3'-UTR of the *icaR* mRNA	This study
pMAD 3xFLAG-icaR	pMAD plasmid containing the allele for insertion of the 3xFLAG at the N-terminus of the IcaR protein	This study
pMAD Δ*SA1387*	pMAD plasmid containing the allele for deletion of the DEAD-box RNA helicase (*S. aureus* N315 ID: SA1387)	This study
pLUG533	pMAD derivative for deletion/replacement of *S. aureus hfq* gene	[2]
pSA14	*E. coli* - *S. aureus* shuttle vector for transcriptional LacZ reporter fusions. The plasmid carries the promoterless *Escherichia coli lacZ* gene downstream from the *Bacillus subtilis spoVG* ribosome binding site.	[3]
pSA14-P*ica*	pSA14 plasmid carrying the *ica* operon promoter region from −422 to +1, where +1 corresponds to the mapped transcriptional start site of *icaADBC* mRNA	This study
pCN40	*E. coli* - *S. aureus* shuttle vector to express genes under the control of the P_{blaZ} constitutive promoter. Low copy number (20 to 25 copies/cell). Em^R	[4]
p^{FLAG}IcaRm	pCN40 plasmid constitutively expressing the N-terminal 3XFLAG tagged IcaR protein from the entire mRNA	This study
p^{FLAG}IcaRmΔ3'UTR	pCN40 plasmid constitutively expressing the N-terminal 3XFLAG tagged IcaR protein from the mRNA carrying a deletion of 331 nt downstream of the stop codon	This study
p^{FLAG}IcaRmΔanti-SD	pCN40 plasmid constitutively expressing the N-terminal 3XFLAG tagged IcaR protein from the mRNA carrying the UCCCCUG deletion	This study
p^{FLAG}IcaRm-SUBST	pCN40 plasmid constitutively expressing the N-terminal 3XFLAG tagged IcaR protein from the mRNA carrying the substitution of the UCCCCUG motif by AGGGGAC	This study
pIcaRm	pCN40 plasmid constitutively expressing the entire *icaR* mRNA molecule	This study
pIcaRmΔ3'UTR	pCN40 plasmid constitutively expressing the *icaR* mRNA carrying a deletion of 331 nt downstream of the stop codon	This study
pIcaRmΔanti-SD	pCN40 plasmid constitutively expressing the entire *icaR* mRNA carrying the UCCCCUG deletion	This study
pIcaRm-SUBST	pCN40 plasmid constitutively expressing the *icaR* mRNA carrying the substitution of the UCCCCUG motif by AGGGGAC	This study
pIcaRm-Compensatory	pCN40 plasmid constitutively expressing the *icaR* mRNA carrying the substitution of the UCCCCUG motif by AGGGGAC and the substitution of the SD region (CAGGGGG) by GTCCCCT	This study
pET-15b RNase III	pET-15b expressing the *S. aureus* rnc gene which encodes the double stranded endoribonuclease RNase III fused to His-tag.	This study
pUT7-*spa*	T7 promoter-*spa* (nts -25 to +200) allowing the in vitro T7 transcription of *S. aureus spa* mRNA containing the whole 5'-UTR and 200 nts of the coding sequence	[5]
pUT7-*icaR*	T7 promoter-*icaR* allowing the *in vitro* T7 transcription of the full-length *icaR* mRNA	This study

[a]References

1. Arnaud M, Chastanet A, Débarbouillé M (2004) New vector for efficient allelic replacement in naturally nontransformable, low-GC-content, Gram-positive bacteria. Applied and Environmental Microbiology 70: 6887–6891.
2. Boisset S, Geissmann T, Huntzinger E, Fechter P, Bendridi N, et al. (2007) *Staphylococcus aureus* RNAIII coordinately represses the synthesis of virulence factors and the transcription regulator Rot by an antisense mechanism. Genes & Development 21: 1353–1366.
3. Joanne P, Falord M, Chesneau O, Lacombe C, Castano S, et al. (2009) Comparative study of two plasticins: specificity, interfacial behavior, and bactericidal activity. Biochemistry 48: 9372–9383.
4. Charpentier E, Anton AI, Barry P, Alfonso B, Fang Y, et al. (2004) Novel cassette-based shuttle vector system for Gram-positive bacteria. Applied and Environmental Microbiology 70: 6076–6085.
5. Huntzinger E, Boisset S, Saveanu C, Benito Y, Geissmann T, et al. (2005) *Staphylococcus aureus* RNAIII and the endoribonuclease III coordinately regulate *spa* gene expression. EMBO J 24: 824–835.
