# Supplementary material for: Base Pairing Interaction between 5′- and 3′-UTRs Controls icaR mRNA Translation in Staphylococcus aureus
Source: PLoS Genet. 2013 Dec 19;9(12):e1004001. doi: 10.1371/journal.pgen.1004001 (PMC3868564; doi:10.1371/journal.pgen.1004001)
Supplement: Table S3 — Oligonucleotides used in this study. (PDF) [file pgen.1004001.s010.pdf]

Table S3. Oligonucleotides used in this study.

Oligonucleotide	Sequence
**mRACE for simultaneous mapping of transcriptional start and stop sites**	
RACE-IcaR-1	TATCATCAAGTGTTGTACCGTCAT
RACE-IcaR-2	TCAAAGATGAAGTGTATTCGCTAC
**Northern blot probes**	
NB-IcaR-Sense	AGCTATATCATCAAGTGTTGTACCGTCATACCCCTTCTCTG
400-Sense	TGTAATTAGATGACAACTTATTCTTTTCAGGGGAAC
RP-T7-Sense-IcaR	TAATACGACTCACTATAGGGTACTTTCTTCCACTGCTCCA
IcaR+1 (BamHI)	*GGATCC*GAAATATTTGTAATTGCA
**Deletion of *icaR* 3'-UTR**	
IcaR-A (EcoRI)	*GAATTC*TTTCAGAGAAGGGGTATGACG
IcaR-B (SalI)	*GTCGAC*CAAAAATTATTTCTTCAAAAATAT
IcaR-C (SalI)	*GTCGAC*TAATTTATTGATAAGCGCCTA
IcaR-D (BamHI)	*GGATCC*AACGACCACAAAACATACACAA
IcaR-E	GAGTAGAAGCAGTATATTTGT
IcaR-F	AAATAGAGTGAAGACACCC
**Deletion of *dbpA* gene**	
SA1387-A (BamHI)	*GGATCC*TCTTATCTTCACCAACATAA
SA1387-B (HindIII)	*AAGCTT*GTGTGTTTTATGTTATTAGG
SA1387-C (HindIII)	*AAGCTT*ATTTGGCCTCCTTATATGT
SA1387-D (BamHI)	*GGATCC*CTTTGAAAGTGAAGGAAGA
SA1387-E	ACAGTAATGTTTACGACTCA
SA1387-F	GTAGTGTATAAGCGACATTA
**mRNA levels quantification by qRT-PCR**	
icaC2.SG-5	TGCGTTAGCAAATGGAGACTATTG
icaC2.SG-3	TGAGAAAGCACTAATCATTTGAATGC
icaR.SG-5	TTCTTATTTGAGTTTATTTTCGACATCG
icaR.SG-3	CATAGAATTTTGCTATCTCTTTACTTAATGATTG
gyrB.SG-5	ACGTATGAAGGTGGTACGCATG
gyrB.SG-3	ACGCGTTAATGCACGTTTGA
**Transcriptional fusions for *icaR* and *icaADBC* promoters**	
icaA-pSA14-Fw (PstI)	*CTGCAG*CGTACATTCTAATATACCT
icaA-pSA14-Rv (BamHI)	*GGATCC*TAAGCCATATGGTAATTGATAG
**Constitutive expression of *icaR* and icaRmΔ3'-UTR mRNAs**	
IcaR+1 (BamHI)	*GGATCC*GAAATATTTGTAATTGCA
IcaR-Term (EcoRI)	*GAATTC*CCTTTTAAAAAGATGTGGGTA
**Constitutive expression of 3XFLAG IcaR protein**	
IcaR+1 (BamHI)	*GGATCC*GAAATATTTGTAATTGCA
IcaR-3XFLAG-DCHA	GACTACAAAGACCATGACGGTGATTATAAAGATCATGATATCGACTACAAAGATGACGACG ATAAAAAGGATAAGATTATTGATAACGCA
IcaR-3XFLAG-IZDA	ATGATCTTTATAATCACCGTCATGGTCTTTGTAGTCCAATTTTTATAACCCCCTACT
IcaR-Term (EcoRI)	*GAATTC*CCTTTTAAAAAGATGTGGGTA
**Constitutive expression of *icaR* mRNA carrying the UCCCCUG motif deletion**	
DEL-AntiRBS-IZDA-2	TGACAACTTATTCTTTTACATTACACTTTTATAATATGTTCAA
DEL-AntiRBS-DCHA	ATGTAAAAGAATAAGTTGTCATCTAATTACA
**Constitutive expression of *icaR* mRNA carrying the UCCCCUG motif substitution**	
SUBST-AntiRBS-IZDA-2	TGACAACTTATTCTTTTGTCCCCTACATTACACTTTTATAATATGTTCAA
SUBST-AntiRBS-DCHA	ATGTAGGGGACAAAAGAATAAGTTGTCATCTAATTACA
**Constitutive expression of *icaR* mRNA carrying the compensatory mutation at the SD region**	
SUBST-RBS-DCHA	CAGGTCCCCTTTATAAAAATTGAAGGATAAG
SUBST-RBS-IZDA	CAATTTTTATAAAGGGGACCTGAAAATTAATCACACTATGTTAC

Continued on following page

Table S3. *Continued*

**Synthesis of the substrates for in vitro assays**	
RNA T7 icaR +1	TAATACGACTCACTATAGGGGAAATATTTGTAATTGCAACTTAATTT
RP 5'UTR icaR RV	CTCTGAAAATAAGGTTATTGCG
RNA T7 3'UTR ucccc Fw	TAATACGACTCACTATAGGGCTTTATATTTGTGAATGGTTAAGT
RP3´UTR ucccc Rv	TTCCATTTCATCTAATTTATGCGG
**Producction of Recombinat RNAse III**	
RNAse III Fw (NdeI)	*GGAATT*CCATATGTCTAAACAAAAGAAAAGTGAG
RNAse III Rv (BamHI)	*CGCGGA*TCCCTATTTAATTTGTTTTAATTGCTTATAG
**mRACE for mapping of processing sites at the 3'-UTR**	
RACE-Frag-400-RT	TTCATCTAATTTATGCGGATTTCCTG
icaR-C (SalI)	*GTCGAC*TAATTTATTGATAAGCGCCTA
**Toeprint**	
IcaR Forward	GGGAAATATTTGTAATTGCAACTTAATTTTCC
IcaR Reverse	TCCAT*GGATCC*AAAAAAGCGCCTATGTCATGATTTAC
T7 5'UTR	TAATACGACTCACTATAGGGAAATATTTGTAATTGCCAACTTAATTT
IcaR 5' rev	ATCATCAAGTGTTGTACCGTCATACCCCTT
T7 3'UTR	TAATACGACTCACTATAGGGCTTTATATTTGTGAATGGTTAAGTTTGTCTTTGAAC
IcaR 3' term	AAAAAGCGCCTATGTCATGATTTACCATCA
spa	TTTGCAGCAGGTGTTACGCC

Italic, restriction enzyme site included in the oligonucleotide. Underlined, T7 promoter
